# Supplementary material for: Matrix games between full siblings in Mendelian populations
Source: PLoS One. 2025 Sep 10;20(9):e0331044. doi: 10.1371/journal.pone.0331044 (PMC12422505; doi:10.1371/journal.pone.0331044)
Supplement: S1 File — (DOCX) [file pone.0331044.s001.docx]

**Matrix games between full siblings in Mendelian populations**

SUPPLEMENTARY INFORMATION

József Garay, Villő Csiszár, Tamás F. Móri, Tamás Varga, András Szilágyi

**SI A. Results on evolutionarily stable genotype distributions (ESGD)**

We remind of the definition of evolutionary stability of a genotype distribution. A genotype distribution $x^{*}\in\text{int}S_{m}$ is evolutionarily stable if

$$\sum_{i} x_{i}^{*}U_{i}\left( x \right)>\bar{U}\left( x \right)$$

provided that $x$ is sufficiently close to $x^{*}$*.* Consequently, $x^{*}\in\text{int}\text{ }S_{m}$ is an ESGD if and only if

$F\left( x^{*},x \right)=\sum_{i} \left( x_{i}^{*}-x_{i} \right)\frac{V_{i}\left( x \right)}{x_{i}}>0,$ $(\mathrm{SI} 1)$

whenever $x$ is close enough to $x^{*}$; that is, there is a $\delta>0$ such that the above inequality holds provided $0<\left\| x^{*}-x \right\|<\delta$. Considering the Taylor expansion of $V_{i}\left( x \right)/{x_{i}}$ at $x^{*}\text{,}$ we have

$\frac{V_{i}\left( x \right)}{x_{i}}=\frac{V_{i}\left( x^{*} \right)}{x_{i}^{*}}+\sum_{j} \varphi_{i,j}\left( x \right)\cdot\left( x_{j}-x_{j}^{*} \right)$ (SI 2)

where $\varphi_{i,j}\left( x \right)=\partial_{x_{j}}\left( V_{i}\left( x \right)/{x_{i}} \right)+\omega_{i,j}\left( x \right)$ with $\lim_{x\to x^{*}} \omega_{i,j}(x)=0 \left( 1\leq i,j\leq m \right)$, and there exist $K$ and $\bar{\delta}>0$, independent of $i$ and $j$, such that $\left| \varphi_{ij}\left( x \right) \right|<K$ $\left( 1\leq i,j\leq m \right)$ for any $x$ with $\left\| x-x^{*} \right\|<\bar{\delta}\text{.}$ Since $S_{m}$ is bounded, there is a $t_{0}$ such that $t\left\| x-x^{*} \right\|<\text{min}\left( \delta,\bar{\delta} \right)$ for every $x\in S_{m}$ and $t\in\left( 0,t_{0} \right]$. Consequently, if $x\in S_{m}$ and $t\in\left( 0,t_{0} \right]$ then

$$0<F\left( x^{*},x^{*}+t\left( x-x^{*} \right) \right)=\sum_{i} t\left( x_{i}^{*}-x_{i} \right)\frac{V_{i}\left( x^{*}+t\left( x-x^{*} \right) \right)}{x_{i}^{*}+t\left( x_{i}-x_{i}^{*} \right)}$$

$$=t\sum_{i} \left( x_{i}^{*}-x_{i} \right)\left( \frac{V_{i}\left( x^{*} \right)}{x_{i}^{*}}+\sum_{j} \varphi_{i,j}\left( x^{*}+t\left( x-x^{*} \right) \right) t\left( x_{j}-{x_{j}}^{*} \right) \right)$$

$$\text{= }t\left( \sum_{i} \left( x_{i}^{*}-x_{i} \right)\frac{V_{i}\left( x^{*} \right)}{x_{i}^{*}}+t\sum_{i,j} \left( x_{i}^{*}-x_{i} \right)\varphi_{i,j}\left( x^{*}+t\left( x-x^{*} \right) \right) \left( x_{j}-x_{j}^{*} \right) \right)\text{.}$$

Dividing by *t* (recall that $t>0$) we get that

$$0<\sum_{i} \left( x_{i}^{*}-x_{i} \right)\frac{V_{i}\left( x^{*} \right)}{x_{i}^{*}}+t\sum_{i,j} \left( x_{i}^{*}-x_{i} \right)\varphi_{i,j}\left( x^{*}+t\left( x-x^{*} \right) \right)\left( x_{j}-x_{j}^{*} \right)$$

for any $t\in\left( 0,t_{0} \right]$. Since the functions $\varphi_{i,j}$ are uniformly bounded on the disk centered at $x^{*}$ with radius $\bar{\delta}$, and the strict inequality above holds for arbitrary small positive $t$, we infer the following Nash equilibrium condition.

**Equilibrium condition** If $x^{*}\in\text{int}\text{ }S_{m}$ is an ESGD and $x$ is an arbitrary state in $S_{m}$ then

$\sum_{i=1}^{m} \left( x_{i}^{*}-x_{i} \right)\frac{V_{i}(x^{*})}{x_{i}^{*}}\geq0.$ (SI 3)

Moreover, one of the characteristic properties of interior Nash equilibria in matrix games is true in this model too, which gives a tool for finding interior Nash equilibria. This is the following.

**Lemma SI.1.** Let $x^{*}\in\text{int}S_{m}$ be an ESGD. Then, for every $i,j\in\left\{ 1,2,\ldots,m \right\}$ we have

$$\frac{V_{i}\left( x^{*} \right)}{x_{i}^{*}}=\frac{V_{j}\left( x^{*} \right)}{x_{j}^{*}}\text{.}$$

*Proof.* The proof follows the usual game theoretical reasoning (cf. (6.14)–(6.15) on p.64 in Hofbauer and Sigmund [1]). Indeed, denote by $e_{i}$ the state $x$ with $x_{i}=1$ and $x_{j}=0$ for $j\neq i\text{,}$ that is, the state in which every individual belongs to genotype $i$. According to the equilibrium condition (SI 3) we have that

$$\sum_{j=1}^{m} x_{j}^{*}\frac{V_{j}\left( x^{*} \right)}{x_{j}^{*}}\geq\sum_{j=1}^{m} e_{i,j}\frac{V_{j}\left( x^{*} \right)}{x_{j}^{*}}=\frac{V_{i}\left( x^{*} \right)}{x_{i}^{*}}$$

where $e_{i,j}$ is the $j$-th coordinate of $e_{i}$, that is, $e_{i,j}=1$ if $j=i$ and $e_{i,j}=0$ if $j\neq i\text{.}$ Multiplying by $x_{i}^{*}$ then summing from $i=1$ to $i=m$ we get

$$\sum_{i=1}^{m} x_{i}^{*}\left( \sum_{j} x_{j}^{*}\frac{V_{j}\left( x^{*} \right)}{x_{j}^{*}} \right)=\sum_{j} x_{j}^{*}\frac{V_{j}\left( x^{*} \right)}{x_{j}^{*}}\geq\sum_{i=1}^{m} x_{i}^{*}\frac{V_{i}\left( x^{*} \right)}{x_{i}^{*}} ,$$

which is possible only if $V_{i}\left( x^{*} \right)/{x_{i}^{*}}=\sum x_{j}^{*}V_{j}\left( x^{*} \right)/{x_{j}^{*}}$ for every $i=1,2,\ldots,m\text{.}$ We have made use of the fact that $\sum_{i} x_{i}^{*}=1.$ ■

We note that Lemma SI.1 implies that an ESGD is a rest point of the dynamics (1), see the next section.

Another consequence of the lemma is a negative semidefiniteness condition (cf. [2]). By inequality (SI 1) and (SI 2), if $x$ is close enough to $x^{*}\text{,}$ then

$$0<\sum_{i} \left( x_{i}^{*}-x_{i} \right)\frac{V_{i}\left( x^{*} \right)}{x_{i}^{*}}+\sum_{i,j} \left( x_{i}^{*}-x_{i} \right)\varphi_{i,j}\left( x \right) \left( x_{j}-x_{j}^{*} \right)$$

holds. On the other hand, we have $\sum\left( x_{i}^{*}-x_{i} \right)V_{i}\left( x^{*} \right)/{x_{i}^{*}}=0$ by Lemma SI.1, therefore this inequality simplifies to

$$0<\sum_{i,j} \left( x_{i}^{*}-x_{i} \right)\varphi_{i,j}\left( x \right) \left( x_{j}-x_{j}^{*} \right)=\sum_{i,j} \left( x_{i}^{*}-x_{i} \right)\partial_{x_{j}}\left( \frac{V_{i}\left( x \right)}{x_{i}} \right)_{x=x^{*}}\left( x_{j}-x_{j}^{*} \right)+\sum_{i,j} \left( x_{i}^{*}-x_{i} \right)\omega_{i,j}\left( x \right) \left( x_{j}-x_{j}^{*} \right)\text{.}$$

Since $\omega_{i,j}\left( x \right)\to0$ as $x\to x^{*}$ we infer that

$$0\leq\sum_{i,j} \left( x_{i}^{*}-x_{i} \right)\partial_{x_{j}}\left( \frac{V_{i}\left( x \right)}{x_{i}} \right)_{x=x^{*}}\left( x_{j}-x_{j}^{*} \right)$$

or

$$0\geq\sum_{i,j} \left( x_{i}-x_{i}^{*} \right)\partial_{x_{j}}\left( \frac{V_{i}\left( x \right)}{x_{i}} \right)_{x=x^{*}}\left( x_{j}-x_{j}^{*} \right),$$

which just means that the matrix $\left[ \left. \partial_{x_{j}}\left( V_{i}\left( x \right)/{x_{i}} \right) \right|_{x=x^{*}} \right]_{i,j}$ is negative semidefinite. (In the case of classical evolutionary matrix games negative definiteness holds because there the functions $\omega_{i,j}$ are identically zero (see Exercise 6.4.3 in Hofbauer and Sigmund [1]).

**SI B. Stability of ESGD with respect to genotype dynamics**

Consider the genotype dynamics (1)

$$\dot{x}_{i}=V_{i}\left( x \right)-x_{i}\sum_{j} V_{j}\left( x \right), i=1,2,\ldots,m.$$

**Theorem SI.1** *Assume that* $x^{*}\in\text{int} S_{m}$ *is an ESGD. Then* $x^{*}$ *is a locally asymptotically stable rest point of genotype dynamics* (1).

*Proof.* The proof follows the proof of Theorem 7.2.4 in Hofbauer and Sigmund [1].

By Lemma SI.1, $x^{*}$ is an interior rest point of the genotype dynamics (1).

We prove that $x^{*}$ asymptotically stable. To see this, consider the function

$$L\left( x \right)=-\sum_{i=1}^{m} x_{i}^{*}\text{ln}\frac{x_{i}}{x_{i}^{*}},$$

which is a Lyapunov function with respect to genotype dynamics (1) at state $x^{*}.$ Here $L\left( x^{*} \right)=0$ and $x^{*}$ is a strict local minimum point of $L$ , see the proof of Theorem 7.2.4 in Hofbauer and Sigmund [1]. On the other hand, the derivative of $L\left( x \right)$ along the differential equation (1) is

$$\frac{d}{dt}L\left( x \right)=-\sum_{i=1}^{m} x_{i}^{*}\frac{\dot{x}_{i}}{x_{i}}=-\sum_{i=1}^{m} x_{i}^{*}\frac{V_{i}\left( x \right)-x_{i}\sum V_{j}\left( x \right)}{x_{i}}$$

$$\text{=}\sum_{j=1}^{m} V_{j}\left( x \right)-\sum_{i=1}^{m} x_{i}^{*}\frac{V_{i}\left( x \right)}{x_{i}}=\sum_{i=1}^{m} \left( x_{i}-x_{i}^{*} \right)\frac{V_{i}\left( x \right)}{x_{i}}<0$$

since $x^{*}$ is an ESGD. This means that $\frac{d}{dt}L\left( x \right)$ is negative definite at $x^{*}.$ By Lyapunov’s theorem on stability, this implies that $x^{*}$ is an asymptotically stable rest point of the genotype dynamics (1), see e.g. Theorem 2.6.1 in Hofbauer and Sigmund [1]; p.194 in Hirsch et al. [3] or Theorem 3.5.1(b) in Kong [4]. ∎

**SI C. Matrix game within a monogamous family**

Let us briefly summarize the considered model we are using. There are $N$ individuals ($N$ is a very large even integer), half of them are male, half of them are female. The proportions of the three genotypes $G_{1}=(\left[ a \right],\left[ a \right]), G_{2}=(\left[ a \right],\left[ A \right]), G_{3}=(\left[ A \right],\left[ A \right])$ are the same among males and females, namely $x_{1}, x_{2}, x_{3}$. They form $N/2$ male-female pairs (families) uniformly at random. Each family produces $n$ offspring according to the Mendelian rules ($n$ is supposed to be constant and even). Then these offspring form $n/2$ pairs uniformly at random, and the pairs play a matrix game with payoff matrix $A=\left( a_{v,w} \right)\in{(0, 1]}^{2\times2}$. If a player uses pure strategy $v$ against an opponent using pure strategy $w$, then its survival probability will be $a_{v,w}$ ($v,w\in\{1, 2\}$). Of course, mixed strategies are also allowed. Suppose genotypes $G_{1}$ and $G_{3}$ always use pure strategies $s_{1}=(1,0)$ and $s_{3}=(0,1)$, resp. The strategy $s_{2}$ of $G_{2}$ depends on the type of inheritance. In the recessive case $s_{2}=s_{3}$, in the dominant case $s_{2}=s_{1}$, while in the intermediate case $s_{2}=\frac{1}{2}\left( s_{1}+s_{3} \right)$. Let $n_{k(i,j)}$ denote the expected number of surviving offspring in a family of type $G_{i}\times G_{j}$ ($i,j,k\in\left\{ 1, 2, 3 \right\}$). Firstly, we are going to compute these numbers.

During the calculus the following elementary facts will be used.

**Fact.** Suppose that in a random experiment there are $m$ possible mutually exclusive outcomes with corresponding probabilities by $\pi_{1}, \ldots, \pi_{m}$, respectively. Let $\xi_{i}$ indicate the number of times outcome number $i$ is observed over $n$ independent trials. Then the joint distribution of the random vector $\left( \xi_{1}, \ldots, \xi_{m} \right)$ is multinomial with parameters $n,\pi_{1}, \ldots, \pi_{m}$. Moreover,

$$\mathbb{E}\left[ \xi_{i}\left( \xi_{i}-1 \right) \right]=n\left( n-1 \right)\pi_{i}^{2}\mathbb{, E}\left( \xi_{i}\xi_{j} \right)=n\left( n-1 \right)\pi_{i}\pi_{j} \left( i\neq j \right).$$

**Family type** $\boldsymbol{G}_{\boldsymbol{1}}\boldsymbol{\times}\boldsymbol{G}_{\boldsymbol{1}}$. Then $m=1$, and $n_{1\left( 11 \right)}=ns_{1}As_{1}$, $n_{2\left( 11 \right)}=n_{3\left( 11 \right)}=0$.

**Family type** $\boldsymbol{G}_{\boldsymbol{1}}\boldsymbol{\times}\boldsymbol{G}_{\boldsymbol{2}}$**.** Then $m=2, \pi_{1}=\pi_{2}=\frac{1}{2}$, and

$n_{1\left( 12 \right)}=\frac{1}{n-1}\left( \mathbb{E}\left[ \xi_{1}\left( \xi_{1}-1 \right) \right]s_{1}As_{1}\mathbb{+E}\left( \xi_{1}\xi_{2} \right)s_{1}As_{2} \right)=\frac{n}{4}\left( s_{1}As_{1}+s_{1}As_{2} \right)$,

$n_{2\left( 12 \right)}=\frac{1}{n-1}\left( \mathbb{E}\left( \xi_{2}\xi_{1} \right)s_{2}As_{1}\mathbb{+E}\left[ \xi_{2}\left( \xi_{2}-1 \right) \right]s_{2}As_{2} \right)=\frac{n}{4}\left( s_{2}As_{1}+s_{2}As_{2} \right)$,

$n_{3(12)}=0$.

**Family type** $\boldsymbol{G}_{\boldsymbol{1}}\boldsymbol{\times}\boldsymbol{G}_{\boldsymbol{3}}$**.** Then $m=1$, and $n_{2\left( 13 \right)}=ns_{2}As_{2}$, $n_{1\left( 13 \right)}=n_{3\left( 13 \right)}=0$.

**Family type** $\boldsymbol{G}_{\boldsymbol{2}}\boldsymbol{\times}\boldsymbol{G}_{\boldsymbol{2}}$**.** Then $m=3$, $\pi_{1}=\pi_{3}=\frac{1}{4}, \pi_{2}=\frac{1}{2}$ , and

$n_{1\left( 22 \right)}=\frac{1}{n-1}\left( \mathbb{E}\left[ \xi_{1}\left( \xi_{1}-1 \right) \right]s_{1}As_{1}\mathbb{+E}\left( \xi_{1}\xi_{2} \right)s_{1}As_{12}\mathbb{+E}\left( \xi_{1}\xi_{3} \right)s_{1}As_{2} \right)$

$=\frac{n}{16}\left( s_{1}As_{1}+2s_{1}As_{2}+s_{1}As_{3} \right)$,

$n_{2\left( 22 \right)}=\frac{1}{n-1} \left( \mathbb{E}\left( \xi_{2}\xi_{1} \right)s_{2}As_{1}+\mathbb{E}\left[ \xi_{2}\left( \xi_{2}-1 \right) \right]s_{2}As_{2}\mathbb{+ E}\left( \xi_{2}\xi_{3} \right)s_{2}As_{3} \right)$

$=\frac{n}{8}\left( s_{2}As_{1}+2s_{2}As_{2}+s_{2}As_{3} \right)$,

$n_{3\left( 22 \right)}=\frac{1}{n-1} \left( \mathbb{E}\left( \xi_{3}\xi_{1} \right)s_{3}As_{1}\mathbb{+E}\left( \xi_{3}\xi_{2} \right)s_{3}As_{2}\mathbb{+E}\left[ \xi_{3}\left( \xi_{3}-1 \right) \right]s_{3}As_{3} \right)$

$=\frac{n}{16}\left( s_{3}As_{1}+2s_{3}As_{2}+s_{3}As_{3} \right)$.

**Family type** $\boldsymbol{G}_{\boldsymbol{2}}\boldsymbol{\times}\boldsymbol{G}_{\boldsymbol{3}}$**.**  Then $m=2, \pi_{1}=\pi_{2}=\frac{1}{2}$, and

$n_{1(23)}=0$,

$n_{2\left( 23 \right)}=\frac{1}{n-1}\left( \mathbb{E}\left[ \xi_{1}\left( \xi_{1}-1 \right) \right]s_{2}As_{2}\mathbb{+E}\left( \xi_{1}\xi_{2} \right)s_{2}As_{3} \right)=\frac{n}{4}\left( s_{2}As_{2}+s_{2}As_{3} \right)$,

$n_{3\left( 23 \right)}=\frac{1}{n-1}\left( \mathbb{E}\left( \xi_{2}\xi_{1} \right)s_{3}As_{2}+\mathbb{E}\left[ \xi_{2}\left( \xi_{2}-1 \right) \right]s_{3}As_{3} \right)=\frac{n}{4}\left( s_{3}As_{2}+s_{3}As_{3} \right)$.

**Family type** $\boldsymbol{G}_{\boldsymbol{3}}\boldsymbol{\times}\boldsymbol{G}_{\boldsymbol{3}}$. Clearly, $n_{3\left( 33 \right)}=ns_{3}As_{3}$, $n_{1\left( 33 \right)}=n_{2\left( 33 \right)}=0$.

For the sake of simplicity let us denote $s_{1}As_{1,}s_{1}As_{2},s_{2}As_{1},$ and $s_{2}As_{2}$ by $a_{11},a_{12},a_{21}$ and $a_{22}$, respectively.

$\left[ a \right]$ *is recessive*

Then $s_{1}As_{2}=a_{12}$, $s_{2}As_{1}=a_{21}$, $s_{2}As_{2}=s_{3}As_{2}=s_{2}As_{3}=a_{22}$. Therefore the number of offspring of a mating pair with genotypes $G_{i}$ and $G_{j}$ is the following.

$\boldsymbol{G}_{\boldsymbol{1}}\boldsymbol{\times}\boldsymbol{G}_{\boldsymbol{1}}$. $n_{1\left( 11 \right)}=na_{11}$, $n_{2\left( 11 \right)}=n_{3\left( 11 \right)}=0$.

$\boldsymbol{G}_{\boldsymbol{1}}\boldsymbol{\times}\boldsymbol{G}_{\boldsymbol{2}}$. $n_{1\left( 12 \right)}=\frac{n}{4}\left( a_{11}+a_{12} \right),$ $n_{2\left( 12 \right)}=\frac{n}{4}\left( a_{21}+a_{22} \right),$ $n_{3(12)}=0.$

$\boldsymbol{G}_{\boldsymbol{1}}\boldsymbol{\times}\boldsymbol{G}_{\boldsymbol{3}}$. $n_{2\left( 13 \right)}=na_{22}$, $n_{1\left( 13 \right)}=n_{3\left( 13 \right)}=0$.

$\boldsymbol{G}_{\boldsymbol{2}}\boldsymbol{\times}\boldsymbol{G}_{\boldsymbol{2}}$. $n_{1\left( 22 \right)}=\frac{n}{16}\left( a_{11}+3a_{12} \right),$ $n_{2\left( 22 \right)}=\frac{n}{8}\left( a_{21}+3a_{22} \right),$ $n_{3\left( 22 \right)}=\frac{n}{16}\left( a_{21}+3a_{22} \right).$

$\boldsymbol{G}_{\boldsymbol{2}}\boldsymbol{\times}\boldsymbol{G}_{\boldsymbol{3}}$. $n_{1(23)}=0, n_{2\left( 23 \right)}=n_{3\left( 23 \right)}=\frac{n}{2}a_{22}.$

$\boldsymbol{G}_{\boldsymbol{3}}\boldsymbol{\times}\boldsymbol{G}_{\boldsymbol{3}}$. $n_{3\left( 33 \right)}=na_{22}$, $n_{1\left( 33 \right)}=n_{2\left( 33 \right)}=0$.

$\left[ a \right]$ *is dominant*

Then $s_{1}As_{2}={s_{2}As_{2}=s_{2}As_{1}=a}_{11}$, $s_{3}As_{2}=a_{21}$, $s_{2}As_{3}=a_{12}$. Therefore the number of offspring of a mating pair with genotypes $G_{i}$ and $G_{j}$ is the following.

$\boldsymbol{G}_{\boldsymbol{1}}\boldsymbol{\times}\boldsymbol{G}_{\boldsymbol{1}}$. $n_{1\left( 11 \right)}=na_{11}$, $n_{2\left( 11 \right)}=n_{3\left( 11 \right)}=0$.

$\boldsymbol{G}_{\boldsymbol{1}}\boldsymbol{\times}\boldsymbol{G}_{\boldsymbol{2}}$. $n_{1\left( 12 \right)}=n_{2\left( 12 \right)}=\frac{n}{2}a_{11},$ $n_{3(12)}=0.$

$\boldsymbol{G}_{\boldsymbol{1}}\boldsymbol{\times}\boldsymbol{G}_{\boldsymbol{3}}$. $n_{2\left( 13 \right)}=na_{11}$, $n_{1\left( 13 \right)}=n_{3\left( 13 \right)}=0$.

$\boldsymbol{G}_{\boldsymbol{2}}\boldsymbol{\times}\boldsymbol{G}_{\boldsymbol{2}}$. $n_{1\left( 22 \right)}=\frac{n}{16}\left( 3a_{11}+a_{12} \right)$, $n_{2\left( 22 \right)}=\frac{n}{8}\left( 3a_{11}+a_{12} \right)$, $n_{3\left( 22 \right)}=\frac{n}{16}\left( 3a_{21}+a_{22} \right).$

$\boldsymbol{G}_{\boldsymbol{2}}\boldsymbol{\times}\boldsymbol{G}_{\boldsymbol{3}}$. $n_{1(23)}=0$, $n_{2\left( 23 \right)}=\frac{n}{4}\left( a_{11}+a_{12} \right)$, $n_{3\left( 23 \right)}=\frac{n}{4}\left( a_{21}+a_{22} \right)$.

$\boldsymbol{G}_{\boldsymbol{3}}\boldsymbol{\times}\boldsymbol{G}_{\boldsymbol{3}}$. $n_{3\left( 33 \right)}=na_{22}$, $n_{1\left( 33 \right)}=n_{2\left( 33 \right)}=0$.

$\left[ a \right]$ *is intermediate*

Then $s_{1}As_{2}=\frac{1}{2}\left( a_{11}+a_{12} \right)$, $s_{3}As_{2}=\frac{1}{2}\left( a_{21}+a_{22} \right)$, $s_{2}As_{1}=\frac{1}{2}\left( a_{11}+a_{21} \right)$, $s_{2}As_{3}=\frac{1}{2}\left( a_{12}+a_{22} \right)$, $s_{2}As_{2}=\frac{1}{4}\left( a_{11}+a_{12}+a_{21}+a_{22} \right)$. Therefore the number of offspring of a mating pair with genotypes $G_{i}$ and $G_{j}$ is the following.

$\boldsymbol{G}_{\boldsymbol{1}}\boldsymbol{\times}\boldsymbol{G}_{\boldsymbol{1}}$. $n_{1\left( 11 \right)}=na_{11}$, $n_{2\left( 11 \right)}=n_{3\left( 11 \right)}=0$.

$\boldsymbol{G}_{\boldsymbol{1}}\boldsymbol{\times}\boldsymbol{G}_{\boldsymbol{2}}$. $n_{1\left( 12 \right)}=\frac{n}{8}\left( 3a_{11}+a_{12} \right)$, $n_{2\left( 12 \right)}=\frac{n}{16}\left( 3a_{11}+a_{12}+3a_{21}+a_{22} \right)$, $n_{3(12)}=0.$

$\boldsymbol{G}_{\boldsymbol{1}}\boldsymbol{\times}\boldsymbol{G}_{\boldsymbol{3}}$. $n_{2\left( 13 \right)}=\frac{n}{4}\left( a_{11}+a_{12}+a_{21}+a_{22} \right)$, $n_{1\left( 13 \right)}=n_{3\left( 13 \right)}=0$.

$\boldsymbol{G}_{\boldsymbol{2}}\boldsymbol{\times}\boldsymbol{G}_{\boldsymbol{2}}$. $n_{1\left( 22 \right)}=\frac{n}{8}\left( a_{11}+a_{12} \right)$, $n_{2\left( 22 \right)}=\frac{n}{8}\left( a_{11}+a_{12}+a_{21}+a_{22} \right)$, and

$n_{3\left( 22 \right)}=\frac{n}{8}\left( a_{21}+a_{22} \right).$

$\boldsymbol{G}_{\boldsymbol{2}}\boldsymbol{\times}\boldsymbol{G}_{\boldsymbol{3}}$. $n_{1(23)}=0$, $n_{2\left( 23 \right)}=\frac{n}{16}\left( a_{11}+3a_{12}+a_{21}+3a_{22} \right)$, $n_{3\left( 23 \right)}=\frac{n}{8}\left( a_{21}+3a_{22} \right)$.

$\boldsymbol{G}_{\boldsymbol{3}}\boldsymbol{\times}\boldsymbol{G}_{\boldsymbol{3}}$. $n_{3\left( 33 \right)}=na_{22}$, $n_{1\left( 33 \right)}=n_{2\left( 33 \right)}=0$.

*Remark*. Another way of computing these quantities can be accomplished on the basis of the following observation. Consider an arbitrary type family and choose a child uniformly at random. Then the type of the chosen child and the type of their opponent in the matrix game are independent. This may seem surprising at first sight, since nobody can play with themselves. However, let the children be numbered from $1$ to $n$ in the order of birth time or in an arbitrary other way; the only requirement is that the ordering is independent of the genotype. Then select the interacting pairs by a draw. Fix a pair, then the types of the players are clearly independent.

**When will genotypes** $\boldsymbol{G}_{\boldsymbol{1}}$ **and** $\boldsymbol{G}_{\boldsymbol{3}}$ **be evolutionarily stable?**

First, we are looking for conditions guaranteeing that the proportion $x_{1}$ of genotype $G_{1}$ increases from generation to generation provided it was sufficiently close to $1$ in the beginning.

Let us apply Theorem 1 of Garay et al. [5] and the subsection Application thereafter. Here we assume that the survival rates of parents (denoted by $q_{k(ij)}$ in that paper) are the same in all family types, thus the change in the relative frequency of the genotype is determined by the juveniles. Therefore we get that $G_{1}$ is evolutionarily stable if any of the following conditions is satisfied.

(c1) $n_{1\left( 11 \right)}-2n_{2\left( 12 \right)}>0$,

(c2) $n_{1\left( 11 \right)}-2n_{2\left( 12 \right)}=0$, $n_{1\left( 11 \right)}-2n_{2\left( 13 \right)}\geq0$, and $2n_{1\left( 12 \right)}-n_{2\left( 22 \right)}-n_{3\left( 22 \right)}>0$,

(c3) $n_{1\left( 11 \right)}-2n_{2\left( 12 \right)}=0$, $n_{1\left( 11 \right)}-2n_{2\left( 13 \right)}<0$, and

$$\frac{n_{3\left( 22 \right)}}{n_{1\left( 11 \right)}}\left( n_{1\left( 11 \right)}-2n_{2\left( 13 \right)} \right)+2n_{1\left( 12 \right)}-n_{2\left( 22 \right)}-n_{3\left( 22 \right)}>0.$$

Note that here (c1) is called the first order condition, while (c2) and (c3) are second order conditions, in the sense of Garay et al [5]. This will become important later.

Since $G_{1}$ and $G_{3}$ play the same role, the conditions of evolutionary stability for $G_{3}$ can be obtained from (c1)–(c3) by interchanging 1 and 3 in the subscripts. Thus, $G_{3}$ is evolutionarily stable if any of the following conditions is satisfied.

(d1) $n_{3\left( 33 \right)}-2n_{2\left( 23 \right)}>0$,

(d2) $n_{3\left( 33 \right)}-2n_{2\left( 23 \right)}=0$, $n_{3\left( 33 \right)}-2n_{2\left( 13 \right)}\geq0$, and ${2n}_{3\left( 23 \right)}-n_{2\left( 22 \right)}-n_{1\left( 22 \right)}>0$,

(d3) $n_{3\left( 33 \right)}-2n_{2\left( 23 \right)}=0$, $n_{3\left( 33 \right)}-2n_{2\left( 13 \right)}<0$, and

$$\frac{n_{1\left( 22 \right)}}{n_{3\left( 33 \right)}}\left( n_{3\left( 33 \right)}-2n_{2\left( 13 \right)} \right)+2n_{3\left( 23 \right)}-n_{2\left( 22 \right)}-n_{1\left( 22 \right)}>0.$$

(In what follows conditions (c…) always refer to genotype $G_{1}$ while conditions (d…) to genotype $G_{3}$.)

Let us plug in here the expressions we got for the quantities $n_{k\left( ij \right)}$.

$\left[ a \right]$ *is recessive*

$n_{1\left( 11 \right)}-2n_{2\left( 12 \right)}=n\left( a_{11}-\frac{1}{2}a_{21}-\frac{1}{2}a_{22} \right)$,

$n_{1\left( 11 \right)}-2n_{2\left( 13 \right)}=n\left( a_{11}-2a_{22} \right)$,

${2n}_{1\left( 12 \right)}-n_{2\left( 22 \right)}-n_{3\left( 22 \right)}=n\left( {\frac{1}{2}a}_{11}+{\frac{1}{2}a}_{12}-\frac{3}{16}a_{21}-\frac{9}{16}a_{22} \right)$,

$\frac{n_{3\left( 22 \right)}}{n_{1\left( 11 \right)}}\left( n_{1\left( 11 \right)}-2n_{2\left( 13 \right)} \right)+2n_{1\left( 12 \right)}-n_{2\left( 22 \right)}-n_{3\left( 22 \right)}$

$=n\left( \frac{a_{21}+3a_{22}}{16 a_{11}}\left( a_{11}-2a_{22} \right)+\frac{1}{2}a_{11}+\frac{1}{2}a_{12}-\frac{3}{16}a_{21}-\frac{9}{16}a_{22} \right)$.

Thus, conditions (c1)–(c3) can be written in the following form.

(c4) ${2a}_{11}>a_{21}+a_{22}$,

(c5) ${2a}_{11}=a_{21}+a_{22}$, $a_{11}\geq2a_{22}$, and ${\frac{1}{2}a}_{11}+{\frac{1}{2}a}_{12}>\frac{3}{16}a_{21}+\frac{9}{16}a_{22}$ ,

(c6) ${2a}_{11}=a_{21}+a_{22}$, $a_{11}<2a_{22}$, and

$\frac{a_{21}+3a_{22}}{16 a_{11}}\left( a_{11}-2a_{22} \right)+\frac{1}{2}a_{11}+\frac{1}{2}a_{12}-\frac{3}{16}a_{21}-\frac{9}{16}a_{22}>0$.

Replace $a_{21}$ with ${2a}_{11}-a_{22}$ in the last inequality of (c5) to obtain $a_{11}+4a_{12}>3a_{22}$. In (c6), after some calculus, the last inequality can be transformed into the more symmetric one $\left( a_{11}+a_{12} \right)\left( a_{21}+a_{22} \right)>\left( a_{11}+a_{22} \right)^{2}$.

Let us turn to $G_{3}$.

$n_{3\left( 33 \right)}-2n_{2\left( 23 \right)}=0$,

$n_{3\left( 33 \right)}-2n_{2\left( 13 \right)}=-na_{22}$,

$2n_{3\left( 23 \right)}-n_{2\left( 22 \right)}-n_{1\left( 22 \right)}= n\left( -\frac{1}{16}a_{11}-\frac{3}{16}a_{12}-\frac{1}{8}a_{21}+\frac{5}{8}a_{22} \right)$,

$\frac{n_{1\left( 22 \right)}}{n_{3\left( 33 \right)}}\left( n_{3\left( 33 \right)}-2n_{2\left( 13 \right)} \right)+2n_{3\left( 23 \right)}-n_{2\left( 22 \right)}-n_{1\left( 22 \right)}$

$=n\left( -\frac{1}{8}a_{11}-\frac{3}{8}a_{12}-\frac{1}{8}a_{21}+\frac{5}{8}a_{22} \right)$.

Thus, conditions (d1)–(d3) can be rewritten in the following form.

(d4) $0>0$,

(d5) $a_{22}\leq0$, and $-\frac{1}{16}a_{11}-\frac{3}{16}a_{12}-\frac{1}{8}a_{21}+\frac{5}{8}a_{22}>0$,

(d6) $a_{22}>0$, and $-\frac{1}{8}a_{11}-\frac{3}{8}a_{12}-\frac{1}{8}a_{21}+\frac{5}{8}a_{22}>0$.

Here (d4) and (d5) cannot hold by supposition.

Consequently, we arrive at the following theorem.

**Theorem SI.2** (recessive case). $G_{1}$ *is evolutionarily stable if the payoff matrix satisfies any of the following conditions.*

(c7) ${2a}_{11}>a_{21}+a_{22}$,

(c8) ${2a}_{11}=a_{21}+a_{22}$, $a_{11}\geq2a_{22}$, *and* $a_{11}+4a_{12}>3a_{22}$

(c9) ${2a}_{11}=a_{21}+a_{22}$, $a_{11}<2a_{22}$, *and* $\left( a_{11}+a_{12} \right)\left( a_{21}+a_{22} \right)>\left( a_{11}+a_{22} \right)^{2}$.

$G_{3}$ *is evolutionarily stable if*

(d7) $a_{22}>\frac{1}{5}\left( a_{11}+3a_{12}+a_{21} \right)$. ∎

*Remark*. The last inequality of (c9) is satisfied if $7a_{11}\leq2a_{12}$, because then $7a_{11}^{2}\leq2a_{11}a_{12}$,

and ${2a}_{11}>a_{22}$, thus

$\left( a_{11}+a_{22} \right)^{2}<9a_{11}^{2}\leq2a_{11}a_{12}+2a_{11}^{2}=2a_{11}\left( a_{12}+a_{11} \right)=\left( a_{11}+a_{12} \right)\left( a_{21}+a_{22} \right)$.

This condition is somewhat stronger, but linear instead of being quadratic, which makes it more convenient to use for constructing numerical examples.

$\left[ a \right]$ *is dominant*

This case could easily be obtained from the recessive case by interchanging $G_{1}$ with $G_{3}$, and 1 with 3 in the subscripts of $n_{k(ij)}$, but here we will simply repeat the computation.

$n_{1\left( 11 \right)}-2n_{2\left( 12 \right)}=0$,

$n_{1\left( 11 \right)}-2n_{2\left( 13 \right)}=-na_{11}$,

$2n_{1\left( 12 \right)}-n_{2\left( 22 \right)}-n_{3\left( 22 \right)}=n\left( \frac{5}{8}a_{11}-\frac{1}{8}a_{12}-\frac{3}{16}a_{21}-\frac{1}{16}a_{22} \right)$,

$\frac{n_{3\left( 22 \right)}}{n_{1\left( 11 \right)}}\left( n_{1\left( 11 \right)}-2n_{2\left( 13 \right)} \right)+2n_{1\left( 12 \right)}-n_{2\left( 22 \right)}-n_{3\left( 22 \right)}$

$= n\left( \frac{5}{8}a_{11}-\frac{1}{8}a_{12}-\frac{3}{8}a_{21}-\frac{1}{8}a_{22} \right)$

Thus, conditions (c1)–(c3) can be written in the following form.

(c10) $0>0$,

(c11) $a_{11}\leq0$, and $\frac{5}{8}a_{11}-\frac{1}{8}a_{12}-\frac{3}{16}a_{21}-\frac{1}{16}a_{22}>0$,

(c12) $a_{11}>0$, and $\frac{5}{8}a_{11}-\frac{1}{8}a_{12}-\frac{3}{8}a_{21}-\frac{1}{8}a_{22}>0$.

Again, (c10) and (c11) cannot hold.

Let us turn to $G_{3}$.

$n_{3\left( 33 \right)}-2n_{2\left( 23 \right)}=n\left( -\frac{1}{2}a_{11}-\frac{1}{2}a_{12}+a_{22} \right)$,

$n_{3\left( 33 \right)}-2n_{2\left( 13 \right)}=n\left( {-2a_{11}+a}_{22} \right)$,

$2n_{3\left( 23 \right)}-n_{2\left( 22 \right)}-n_{1\left( 22 \right)}= n\left( -\frac{9}{16}a_{11}-\frac{3}{16}a_{12}+\frac{1}{2}a_{21}+\frac{1}{2}a_{22} \right)$,

$\frac{n_{1\left( 22 \right)}}{n_{3\left( 33 \right)}}\left( n_{3\left( 33 \right)}-2n_{2\left( 13 \right)} \right)+2n_{3\left( 23 \right)}-n_{2\left( 22 \right)}-n_{1\left( 22 \right)}$

$=n\left( \frac{3a_{11}+a_{12}}{16 a_{22}}\left( a_{22}-2a_{11} \right)-\frac{9}{16}a_{11}-\frac{3}{16}a_{12}+\frac{1}{2}a_{21}+\frac{1}{2}a_{22} \right)$.

Therefore, conditions (d1)–(d3) can be written in the following form.

(d8) ${2a}_{22}>a_{11}+a_{12}$,

(d9) ${2a}_{22}=a_{11}+a_{12}$, and $a_{22}\geq2a_{11}$, and $a_{22}+4a_{21}>3a_{11}$,

(d10) ${2a}_{22}=a_{11}+a_{12}$, $a_{22}<2a_{11}$, and

$\frac{3a_{11}+a_{12}}{16 a_{22}}\left( a_{22}-2a_{11} \right)-\frac{9}{16}a_{11}-\frac{3}{16}a_{12}+\frac{1}{2}a_{21}+\frac{1}{2}a_{22}>0$.

In (d10), after some calculus, the last inequality can be transformed into

$\left( a_{11}+a_{12} \right)\left( a_{21}+a_{22} \right)>\left( a_{11}+a_{22} \right)^{2}$.

Thus, we arrive at the following result.

**Theorem SI.3** (dominant case). $G_{1}$ *is evolutionarily stable if the payoff matrix satisfies*

(c13) $a_{11}>\frac{1}{5}\left( a_{12}+3a_{21}+a_{22} \right)$*.*

$G_{3}$ *is evolutionarily stable if any of the following three conditions is satisfied:*

(d11) ${2a}_{22}>a_{11}+a_{12}$,

(d12) ${2a}_{22}=a_{11}+a_{12}$, $a_{22}\geq2a_{11}$, *and* $a_{22}+4a_{21}>3a_{11}$,

(d13) ${2a}_{22}=a_{11}+a_{12}$, $a_{22}<2a_{11}$, *and*

$\left( a_{11}+a_{12} \right)\left( a_{21}+a_{22} \right)>\left( a_{11}+a_{22} \right)^{2}$. ∎

*Remark.* The last inequality of (d13) follows from the first equation and the second inequality there plus the linear inequality $7a_{22}\leq2a_{21}$. This can be proved e.g. by interchanging the subscripts 1 and 2 everywhere in the Remark after Theorem SI.2.

$\left[ a \right]$ *is intermediate*

$n_{1\left( 11 \right)}-2n_{2\left( 12 \right)}=n\left( \frac{5}{8}a_{11}-\frac{1}{8}a_{12}-\frac{3}{8}a_{21}-\frac{1}{8}a_{22} \right)$,

$n_{1\left( 11 \right)}-2n_{2\left( 13 \right)}=\frac{n}{2}\left( a_{11}-a_{12}-a_{21}-a_{22} \right)$,

${2n}_{1\left( 12 \right)}-n_{2\left( 22 \right)}-n_{3\left( 22 \right)}=n\left( \frac{5}{8}a_{11}+\frac{1}{8}a_{12}-\frac{1}{4}a_{21}-\frac{1}{4}a_{22} \right)$,

$\frac{n_{3\left( 22 \right)}}{n_{1\left( 11 \right)}}\left( n_{1\left( 11 \right)}-2n_{2\left( 13 \right)} \right)+2n_{1\left( 12 \right)}-n_{2\left( 22 \right)}-n_{3\left( 22 \right)}$

$= n\left( \frac{a_{21}+a_{22}}{16a_{11}}\left( a_{11}-a_{12}-a_{21}-a_{22} \right)+\frac{5}{8}a_{11}+\frac{1}{8}a_{12}-\frac{1}{4}a_{21}-\frac{1}{4}a_{22} \right)$.

Thus, conditions (c1)–(c3) can be written in the following form.

(c14) $a_{11}>\frac{1}{5}\left( a_{12}+3a_{21}+a_{22} \right)$,

(c15) $a_{11}=\frac{1}{5}\left( a_{12}+3a_{21}+a_{22} \right)$, $a_{11}\geq a_{12}+a_{21}+a_{22}$, and

$\frac{5}{8}a_{11}+\frac{1}{8}a_{12}-\frac{1}{4}a_{21}-\frac{1}{4}a_{22}>0$,

(c16) $a_{11}=\frac{1}{5}\left( a_{12}+3a_{21}+a_{22} \right)$, $a_{11}<a_{12}+a_{21}+a_{22}$, and

$\frac{a_{21}+a_{22}}{16a_{11}}\left( a_{11}-a_{12}-a_{21}-a_{22} \right)+\frac{5}{8}a_{11}+\frac{1}{8}a_{12}-\frac{1}{4}a_{21}-\frac{1}{4}a_{22}>0$.

Condition (c15) contains a contradiction, so it cannot hold. In (c16), the middle inequality follows from the first equation. The last inequality of (c16) can be transformed into the following one: $4a_{11}\left( a_{12}+a_{21} \right)>\left( a_{21}+a_{22} \right)\left( a_{11}+a_{12}+a_{21}+a_{22} \right)$.

Let us turn to $G_{3}$. By symmetry, the analogues of (c14)–(c16) can be obtained by interchanging 1 and 2 in the subscripts, but they can also be derived directly.

$n_{3\left( 33 \right)}-2n_{2\left( 23 \right)}=n\left( -\frac{1}{8}a_{11}-\frac{3}{8}a_{12}-\frac{1}{8}a_{21}+\frac{5}{8}a_{22} \right)$,

$n_{3\left( 33 \right)}-2n_{2\left( 13 \right)}=\frac{n}{2}\left( {-a_{11}-a_{12}-a_{21}+a}_{22} \right)$,

${2n}_{3\left( 23 \right)}-n_{2\left( 22 \right)}-n_{1\left( 22 \right)}= n\left( -\frac{1}{4}a_{11}-\frac{1}{4}a_{12}+\frac{1}{8}a_{21}+\frac{5}{8}a_{22} \right)$,

$\frac{n_{1\left( 22 \right)}}{n_{3\left( 33 \right)}}\left( n_{3\left( 33 \right)}-2n_{2\left( 13 \right)} \right)+2n_{3\left( 23 \right)}-n_{2\left( 22 \right)}-n_{1\left( 22 \right)}$

$=n\left( \frac{a_{11}+a_{12}}{16 a_{22}}\left( {-a_{11}-a_{12}-a_{21}+a}_{22} \right)-\frac{1}{4}a_{11}-\frac{1}{4}a_{12}+\frac{1}{8}a_{21}+\frac{5}{8}a_{22} \right)$.

Hence, conditions (d1)–(d3) yield the following ones.

(d14) $a_{22}>\frac{1}{5}\left( a_{11}+3a_{12}+a_{21} \right)$,

(d15) $a_{22}=\frac{1}{5}\left( a_{11}+3a_{12}+a_{21} \right)$, $a_{22}\geq{a_{11}+a}_{12}+a_{21}$, and

$-\frac{1}{4}a_{11}-\frac{1}{4}a_{12}+\frac{1}{8}a_{21}+\frac{5}{8}a_{22}>0$,

(d16) $a_{22}=\frac{1}{5}\left( a_{11}+3a_{12}+a_{21} \right)$, $a_{22}<{a_{11}+a}_{12}+a_{21}$, and

$\frac{a_{11}+a_{12}}{16 a_{22}}\left( {-a_{11}-a_{12}-a_{21}+a}_{22} \right)-\frac{1}{4}a_{11}-\frac{1}{4}a_{12}+\frac{1}{8}a_{21}+\frac{5}{8}a_{22}>0$.

Condition (d15) cannot hold. In (d16), the middle inequality already follows from the first equation. The last inequality of (d16) can be transformed into the following one: $4a_{22}\left( a_{12}+a_{21} \right)>\left( a_{11}+a_{12} \right)\left( a_{11}+a_{12}+a_{21}+a_{22} \right)$.

All these imply the following theorem.

**Theorem SI.4**. (intermediate case). $G_{1}$ *is evolutionarily stable if either*

(c17) $a_{11}>\frac{1}{5}\left( a_{12}+3a_{21}+a_{22} \right)$,

*or*

(c18) $a_{11}=\frac{1}{5}\left( a_{12}+3a_{21}+a_{22} \right)$, *and*

$4a_{11}\left( a_{12}+a_{21} \right)>\left( a_{21}+a_{22} \right)\left( a_{11}+a_{12}+a_{21}+a_{22} \right).$

*Similarly,* $G_{3}$ *is evolutionarily stable if either*

(d17) $a_{22}>\frac{1}{5}\left( a_{11}+3a_{12}+a_{21} \right)$,

*or*

(d18) $a_{22}=\frac{1}{5}\left( a_{11}+3a_{12}+a_{21} \right)$, *and*

$4a_{22}\left( a_{12}+a_{21} \right)>\left( a_{11}+a_{12} \right)\left( a_{11}+a_{12}+a_{21}+a_{22} \right)$. ∎

*Remark.* The last inequality of (c18) can be replaced by the stronger but linear inequality $2a_{12}\geq a_{21}+3a_{22}.$ Indeed, then $4a_{12}+3a_{21}-a_{22}\geq5a_{21}+5a_{22}$. Let us multiply both sides by $\frac{1}{5}\left( a_{12}+a_{21}+a_{22} \right)$ to get

$$a_{11}\left( 4a_{12}+3a_{21}-a_{22} \right)>\frac{1}{5}\left( a_{12}+a_{21}+a_{22} \right)\left( 4a_{12}+3a_{21}-a_{22} \right)\geq\left( a_{12}+a_{21}+a_{22} \right)\left( a_{21}+a_{22} \right),$$

which is tantamount to $4a_{11}\left( a_{12}+a_{21} \right)>\left( a_{21}+a_{22} \right)\left( a_{11}+a_{12}+a_{21}+a_{22} \right)$.

Similarly, the last inequality of (d18) can be replaced by $2a_{21}\geq3a_{11}+a_{12}$.

**Conditions implying or making it likely that** $\boldsymbol{G}_{\boldsymbol{1}}$ **or** $\boldsymbol{G}_{\boldsymbol{3}}$ **possesses the repellor property**

Evolutionary stability of $G_{1}$ and $G_{3}$ means that the vertices $(1,0,0)$ and $(0,0,1)$ are attractors, respectively. On the other hand, if in the first order condition the inequality holds in the opposite direction, the corresponding vertex becomes a repellor (see Garay et al. [5]).

Thus, $(1,0,0)$ is a repellor if

(c19) $n_{1\left( 11 \right)}-2n_{2\left( 12 \right)}<0$.

Similarly, $(0,0,1)$ is a repellor if

(d19) $n_{3\left( 33 \right)}-2n_{2\left( 23 \right)}<0.$

$\left[ a \right]$ *is recessive*

In terms of the payoff matrix, (c19) gives

(c20) ${2a}_{11}<a_{21}+a_{22}$.

As we have already seen, $n_{3\left( 33 \right)}-2n_{2\left( 23 \right)}=0$, hence (d19) can never hold. Thus in this way we cannot find any condition that is sufficient for $(0,0,1)$ to be a repellor. However, our simulations support that reversing the last strict inequality in a second order condition for attractors leads to a condition that produces repellors in many practical cases. In the following, we take the second-order conditions above one by one. Though the conditions obtained in this way are not necessarily sufficient for the vertices $(1,0,0)$ and $(0,0,1)$ to be repellors, but they might be useful for finding appropriate numerical examples. We are going to do this after we have finished with the first order conditions.

Thus we only get the following result.

**Theorem SI.5.** (recessive case) *Vertex* $(1,0,0)$ *is a repellor if* ${2a}_{11}<a_{21}+a_{22}$. ∎

$\left[ a \right]$ *is dominant*

As we have already seen, $n_{1\left( 11 \right)}-2n_{2\left( 12 \right)}=0$. Therefore we cannot infer that vertex $(1,0,0)$ is a repellor. Condition (d19) can be written in the following form.

(d20) ${2a}_{22}<a_{11}+a_{12}$.

Thus we arrive at the following theorem.

**Theorem SI.6.** (dominant case) *Vertex* $(0,0,1)$ *is a repellor if* ${2a}_{22}<a_{11}+a_{12}$. ∎

$\left[ a \right]$ *is intermediate*

In terms of the payoff matrix, (c19) reads as

(c21) $a_{11}<\frac{1}{5}\left( a_{12}+3a_{21}+a_{22} \right)$.

Turning to $(0,0,1)$, we similarly have

(d21) $a_{22}<\frac{1}{5}\left( a_{11}+3a_{12}+a_{21} \right).$

Thus we get the following theorem.

**Theorem SI.7.** (intermediate case)

*Vertex* $(1,0,0)$ *is a repellor if* $a_{11}<\frac{1}{5}\left( a_{12}+3a_{21}+a_{22} \right)$*.*

*Vertex* $(0,0,1)$ *is a repellor if* $a_{22}<\frac{1}{5}\left( a_{11}+3a_{12}+a_{21} \right)$. ∎

Next, we turn to the second order conditions so as to obtain conditions that make it likely, though not prove, that the vertex in question is a repellor. Starting from (c2) and (c3) we can say that vertex $(1,0,0)$ may be a repellor if either

(c22) $n_{1\left( 11 \right)}-2n_{2\left( 12 \right)}=0$, $n_{1\left( 11 \right)}-2n_{2\left( 13 \right)}\geq0$, and ${2n}_{1\left( 12 \right)}-n_{2\left( 22 \right)}-n_{3\left( 22 \right)}<0$,

or

(c23) $n_{1\left( 11 \right)}-2n_{2\left( 12 \right)}=0$, $n_{1\left( 11 \right)}-2n_{2\left( 13 \right)}<0$, and

$$\frac{n_{3\left( 22 \right)}}{n_{1\left( 11 \right)}}\left( n_{1\left( 11 \right)}-2n_{2\left( 13 \right)} \right)+2n_{1\left( 12 \right)}-n_{2\left( 22 \right)}-n_{3\left( 22 \right)}<0.$$

Similarly, by (d2) and (d3), vertex $(0,0,1)$ may be a repellor if either

(d22) $n_{3\left( 33 \right)}-2n_{2\left( 23 \right)}=0$, $n_{3\left( 33 \right)}-2n_{2\left( 13 \right)}\geq0$, and ${2n}_{3\left( 23 \right)}-n_{2\left( 22 \right)}-n_{1\left( 22 \right)}<0$,

or

(d23) $n_{3\left( 33 \right)}-2n_{2\left( 23 \right)}=0$, $n_{3\left( 33 \right)}-2n_{2\left( 13 \right)}<0$, and

$$\frac{n_{1\left( 22 \right)}}{n_{3\left( 33 \right)}}\left( n_{3(33)}-2n_{2\left( 13 \right)} \right)+2n_{3\left( 23 \right)}-n_{2\left( 22 \right)}-n_{1\left( 22 \right)}<0.$$

$\left[ a \right]$ *is recessive*

In terms of the payoff matrix, (c22) can be rewritten as

${2a}_{11}=a_{21}+a_{22}$, $a_{11}\geq2a_{22}$, and $a_{11}+4a_{12}<3a_{22}$.

Furthermore, (c23) yields the following condition.

${2a}_{11}=a_{21}+a_{22}$, $a_{11}<2a_{22}$, and $\left( a_{11}+a_{12} \right)\left( a_{21}+a_{22} \right)<\left( a_{11}+a_{22} \right)^{2}$.

As we have already seen, $n_{3\left( 33 \right)}-2n_{2\left( 13 \right)}<0$, hence (d22) cannot hold. From (d23) we get

$a_{22}<\frac{1}{5}\left( a_{11}+3a_{12}+a_{21} \right).$

**Hint SI.1** (recessive case)

*Vertex* $(1,0,0)$ *is suspected of being a repellor if either*

(c24) ${2a}_{11}=a_{21}+a_{22}$, $a_{11}\geq2a_{22}$, *and* $a_{11}+4a_{12}<3a_{22}$,

*or*

(c25) ${2a}_{11}=a_{21}+a_{22}$, $a_{11}<2a_{22}$, *and* $\left( a_{11}+a_{12} \right)\left( a_{21}+a_{22} \right)<\left( a_{11}+a_{22} \right)^{2}$.

*Vertex* $(0,0,1)$ *is suspected of being a repellor if*

(d24) $a_{22}<\frac{1}{5}\left( a_{11}+3a_{12}+a_{21} \right).$ ∎

*Remark.* The last inequality of (c25) is satisfied if $3a_{22}\geq a_{11}+4a_{12}$, because then

$a_{11}+a_{12}=\frac{1}{4}\left( 3a_{11}+a_{11}+4a_{12} \right)\leq\frac{3}{4}\left( a_{11}+a_{22} \right)$,

$\frac{3}{2}a_{11}=a_{11}+\frac{1}{2}a_{11}<a_{11}+a_{22}$,

therefore

$\left( a_{11}+a_{12} \right)\left( a_{21}+a_{22} \right)=2a_{11}\left( a_{11}+a_{12} \right)\leq\frac{3}{2}a_{11}\left( a_{11}+a_{22} \right)<\left( a_{11}+a_{22} \right)^{2}$.

$\left[ a \right]$ *is dominant*

Now (c22) and (c23) take the following form.

(c26) $a_{11}\leq0$, and $\frac{5}{8}a_{11}-\frac{1}{8}a_{12}-\frac{3}{16}a_{21}-\frac{1}{16}a_{22}<0$,

(c27) $a_{11}>0$, and $\frac{5}{8}a_{11}-\frac{1}{8}a_{12}-\frac{3}{8}a_{21}-\frac{1}{8}a_{22}<0$.

Clearly, (c26) cannot hold, by supposition.

Let us turn to the vertex $(0,0,1)$. From (d22) and (d23) we get

${2a}_{22}=a_{11}+a_{12}$, $a_{22}\geq2a_{11}$, and $a_{22}+4a_{21}<3a_{11}$,

and

${2a}_{22}=a_{11}+a_{12}$, $a_{22}<2a_{11}$, and $\left( a_{11}+a_{12} \right)\left( a_{21}+a_{22} \right)<\left( a_{11}+a_{22} \right)^{2}$,

resp. Thus, we have the following recommendation.

**Hint SI.2** (dominant case)

*Vertex* $(1,0,0)$ *is suspected of being a repellor if*

(c28) $a_{11}<\frac{1}{5}\left( a_{12}+3a_{21}+a_{22} \right)$.

*Vertex* $(0,0,1)$ *is suspected of being a repellor if either*

(d25) ${2a}_{22}=a_{11}+a_{12}$, $a_{22}\geq2a_{11}$, *and* $a_{22}+4a_{21}<3a_{11}$,

*or*

(d26) ${2a}_{22}=a_{11}+a_{12}$, $a_{22}<2a_{11}$, *and* $\left( a_{11}+a_{12} \right)\left( a_{21}+a_{22} \right)<\left( a_{11}+a_{22} \right)^{2}$*.* ∎

*Remark.* Similarly to (c25), the last inequality of (d26) is satisfied if $3a_{11}\geq a_{22}+4a_{21}$, because then $a_{21}+a_{22}\leq\frac{3}{4}\left( a_{11}+a_{22} \right)$, and $\frac{3}{2}a_{22}<a_{11}+a_{22}$, hence

$\left( a_{11}+a_{12} \right)\left( a_{21}+a_{22} \right)=2a_{22}\left( a_{21}+a_{22} \right)\leq\frac{3}{2}a_{22}\left( a_{11}+a_{22} \right)<\left( a_{11}+a_{22} \right)^{2}$.

$\left[ a \right]$ *is intermediate*

Then (c22) and (c23) lead to the following conditions.

(c29) $a_{11}=\frac{1}{5}\left( a_{12}+3a_{21}+a_{22} \right)$, $a_{11}\geq a_{12}+a_{21}+a_{22}$, and

$-\frac{1}{4}a_{11}-\frac{1}{4}a_{12}+\frac{1}{8}a_{21}+\frac{5}{8}a_{22}<0$.

(c30) $a_{11}=\frac{1}{5}\left( a_{12}+3a_{21}+a_{22} \right)$, $a_{11}<a_{12}+a_{21}+a_{22}$, and $4a_{11}\left( a_{12}+a_{21} \right)<\left( a_{21}+a_{22} \right)\left( a_{11}+a_{12}+a_{21}+a_{22} \right)$.

Condition (c29) is still contradictory. In (c30), the first equation implies the next inequality.

Turning to (d22) and (d23) we get

(d27) $a_{22}=\frac{1}{5}\left( a_{11}+3a_{12}+a_{21} \right)$, $a_{22}\geq{a_{11}+a}_{12}+a_{21}$, and

$-\frac{1}{4}a_{11}-\frac{1}{4}a_{12}+\frac{1}{8}a_{21}+\frac{5}{8}a_{22}<0$.

(d28) $a_{22}=\frac{1}{5}\left( a_{11}+3a_{12}+a_{21} \right)$, and $a_{22}<{a_{11}+a}_{12}+a_{21}$ and

$4a_{22}\left( a_{12}+a_{21} \right)<\left( a_{11}+a_{12} \right)\left( a_{11}+a_{12}+a_{21}+a_{22} \right)$.

Again, (d27) cannot hold, because the entries of the payoff matrix are all positive. The middle inequality of (d28) already follows from the first equation.

**Hint SI.3** (intermediate case)

*Vertex* $(1,0,0)$ *is suspected of being a repellor if*

(c30) $a_{11}=\frac{1}{5}\left( a_{12}+3a_{21}+a_{22} \right)$, *and*

$4a_{11}\left( a_{12}+a_{21} \right)<\left( a_{21}+a_{22} \right)\left( a_{11}+a_{12}+a_{21}+a_{22} \right)$.

*Vertex* $(0,0,1)$ *is suspected of being a repellor if*

(d28) $a_{22}=\frac{1}{5}\left( a_{11}+3a_{12}+a_{21} \right)$, *and*

$4a_{22}\left( a_{12}+a_{21} \right)<\left( a_{11}+a_{12} \right)\left( a_{11}+a_{12}+a_{21}+a_{22} \right)$. ∎

*Remark.* The last inequality of (c30) is satisfied if $3a_{12}+a_{21}\leq2a_{22}$, because then

$12a_{12}+4a_{21}\leq8a_{22}$, thus $3\left( 4a_{12}+3a_{21}-a_{22} \right)\leq5\left( a_{21}+a_{22} \right)$. Multiply both sides with $\frac{1}{5}\left( a_{12}+a_{21}+a_{22} \right)$ to get

$\frac{3}{5}\left( a_{12}+a_{21}+a_{22} \right)\left( 4a_{12}+3a_{21}-a_{22} \right)\leq\left( a_{21}+a_{22} \right)\left( a_{12}+a_{21}+a_{22} \right)$,

The left hand side, either positive or negative, can be replaced with $a_{11}\left( 4a_{12}+3a_{21}-a_{22} \right)$,

and thus the inequality becomes strict. Hence

$a_{11}\left( 4a_{12}+3a_{21}-a_{22} \right)<\left( a_{21}+a_{22} \right)\left( a_{12}+a_{21}+a_{22} \right)$,

or equivalently,

$4a_{11}\left( a_{12}+a_{21} \right)<\left( a_{21}+a_{22} \right)\left( a_{11}+a_{12}+a_{21}+a_{22} \right)$.

Similarly, the last inequality (d28) is satisfied if $a_{12}+3a_{21}\leq2a_{11}$. This can be obtained from the lines above by interchanging 1 and 2 in the subscripts.

**SI D. How many identical copy do a focal gene produce?**

1. First, let the focal gene be $[a]$. Let us select a gene uniformly at random from all paternal $[a]$ genes (by symmetry, we can confine ourselves to paternal genes). Then it is
   1. in an individual of genotype $G_{1}$ with probability $\frac{2x_{1}}{2x_{1}+x_{2}}$. Then his spouse is
      1. of genotype $G_{1}$ too with probability $x_{1}$. In this case all offspring are of the same genotype and half of the surviving offspring inherit the focal gene on average: $\frac{1}{2}n_{1(11)}$.
      2. of genotype $G_{2}$ with probability $x_{2}$. In this case again half of the surviving offspring inherit the focal gene on average: $\frac{1}{2}\left( n_{1(12)}+n_{2(12)} \right)$.
      3. of genotype $G_{3}$ with probability $x_{3}$. In this case again half of the surviving offspring inherit the focal gene on average: $\frac{1}{2}n_{2(13)}$.
   2. in an individual of genotype $G_{2}$ with probability $\frac{x_{2}}{2x_{1}+x_{2}}$. Then his spouse is
      1. of genotype $G_{1}$ with probability $x_{1}$. In this case all type $G_{1}$ offspring inherit the focal gene and only they do: $n_{1(12)}$.
      2. of genotype $G_{2}$ with probability $x_{2}$. Again, in this case all type $G_{1}$ offspring inherit the focal gene, but there can be type $G_{2}$ (and even type $G_{3}$) offspring too. The latter ones do not inherit the focal gene, while about half of the the former ones do: $n_{1(22)}+\frac{1}{2}n_{2(22)}$.
      3. of genotype $G_{3}$ with probability $x_{3}$. In this case all type $G_{2}$ offspring inherit the focal gene and only they do: $n_{2(23)}$.

Thus the average number of identical copies of the focal $[a]$ gene in the progeny is equal to

$$W_{a}\left( x \right):=\frac{1}{2x_{1}+x_{2}}\left[ x_{1}^{2}n_{1\left( 11 \right)}+x_{1}x_{2}\left( n_{1\left( 12 \right)}+n_{2\left( 12 \right)} \right)+x_{1}x_{3}n_{2\left( 13 \right)}+x_{1}x_{2}n_{1\left( 12 \right)}+x_{2}^{2}\left( n_{1\left( 22 \right)}+\frac{1}{2} n_{2\left( 22 \right)} \right)+x_{2}x_{3}n_{2\left( 23 \right)} \right]$$

$$=\frac{1}{2x_{1}+x_{2}}\left[ x_{1}^{2}n_{1\left( 11 \right)}+x_{1}x_{2}\left( {2n}_{1\left( 12 \right)}+n_{2\left( 12 \right)} \right)+x_{1}x_{3}n_{2\left( 13 \right)}+x_{2}^{2}\left( n_{1\left( 22 \right)}+\frac{1}{2} n_{2\left( 22 \right)} \right)+x_{2}x_{3}n_{2\left( 23 \right)} \right].$$

1. The average number of identical copies of a focal $[A]$ gene in the progeny can be computed in a similar way. The focal gene is
   1. in an individual of genotype $G_{2}$ with probability $\frac{x_{2}}{2x_{1}+x_{2}}$. Then his spouse is
      1. of genotype $G_{1}$ with probability $x_{1}$. In this case all type $G_{2}$ offspring inherit the focal gene and only they do: $n_{2(12)}$.
      2. of genotype $G_{2}$ with probability $x_{2}$. In this case all type $G_{3}$ offspring inherit the focal gene, but there can be type $G_{2}$ (and even type $G_{1}$) offspring too. The latter ones do not inherit the focal gene, while about half of the former ones do:$n_{3(22)}+\frac{1}{2}n_{2(22)}$.
      3. of genotype $G_{3}$ with probability $x_{3}$. In this case all type $G_{3}$ offspring inherit the focal gene and only they do: $n_{3(23)}$
   2. in an individual of genotype $G_{3}$ with probability $\frac{2x_{3}}{x_{2}+2x_{3}}$. Then his spouse is
      1. of genotype $G_{1}$ with probability $x_{1}$. In this case half of the surviving offspring inherit the focal gene on average: $\frac{1}{2}n_{2(13)}$.
      2. of genotype $G_{2}$ with probability $x_{2}$. In this case again half of the surviving offspring inherit the focal gene on average: $\frac{1}{2}\left( n_{2(23)}+n_{3(23)} \right)$.
      3. of genotype $G_{3}$ too with probability $x_{3}$. In this case all offspring are of the same genotype and half of the surviving offspring inherit the focal gene on average: $\frac{1}{2}n_{3(33)}$.

Thus the average number of identical copies of the focal $[A]$ gene in the progeny is equal to

$$W_{A}\left( x \right):=\frac{1}{x_{2}+2x_{3}}\left[ x_{1}x_{2}n_{2\left( 12 \right)}+x_{2}^{2}\left( n_{3\left( 22 \right)}+\frac{1}{2} n_{2\left( 22 \right)} \right)+x_{2}x_{3}n_{3\left( 23 \right)}+x_{1}x_{3}n_{2\left( 13 \right)}+x_{2}x_{3}\left( n_{2\left( 23 \right)}+n_{3\left( 23 \right)} \right)+x_{3}^{2}n_{3\left( 33 \right)} \right]$$

$$=\frac{1}{x_{2}+2x_{3}}\left[ x_{1}x_{2}n_{2\left( 12 \right)}+x_{2}^{2}\left( n_{3\left( 22 \right)}+\frac{1}{2} n_{2\left( 22 \right)} \right)+x_{1}x_{3}n_{2\left( 13 \right)}+x_{2}x_{3}\left( n_{2\left( 23 \right)}+2n_{3\left( 23 \right)} \right)+x_{3}^{2}n_{3\left( 33 \right)} \right].$$

Note that these results can be obtained in another way. Denoting by $V_{i}(x)$ the number of individuals of genotype $G_{i}$ in the next generation we clearly have

$$W_{a}\left( x \right)=\frac{{2V}_{1}\left( x \right)+V_{2}\left( x \right)}{N\left( 2x_{1}+x_{2} \right)}, W_{A}\left( x \right)=\frac{V_{2}\left( x \right)+{2V}_{3}\left( x \right)}{N\left( x_{2}+2x_{3} \right)} .$$

The denominator contains the total number of genes in the present generation that are of the same type as the focal one, while in the numerator there is the same quantity with respect to the next generation. This is the average number of identical copies of a focal gene in the progeny, indeed.

Obviously,

$$V_{1}\left( x \right)=\frac{N}{2}\left( x_{1}^{2}n_{1(11)}+2x_{1}x_{2}n_{1(12)}+x_{2}^{2}n_{1(22)} \right),$$

$$V_{2}\left( x \right)=\frac{N}{2}\left( 2x_{1}x_{2}n_{2(12)}+2x_{1}x_{3}n_{2(13)}+x_{2}^{2}n_{2(22)}+2x_{2}x_{3}n_{2(23)} \right),$$

$$V_{3}\left( x \right)=\frac{N}{2}\left( x_{2}^{2}n_{3(22)}+2x_{2}x_{3}n_{3(23)}+x_{3}^{2}n_{3(33)} \right).$$

**Theorem SI.8.** *Suppose* $x^{*}=\left( x_{1}^{*},x_{2}^{*},x_{3}^{*} \right)$ *is an ESGD. Then* $W_{a}\left( x^{*} \right)=W_{A}\left( x^{*} \right)$.

*Proof*. By Lemma SI.1 we have

$$V_{1}\left( x^{*} \right)=\frac{x_{1}^{*}}{x_{2}^{*}}V_{2}\left( x^{*} \right), V_{3}\left( x^{*} \right)=\frac{x_{3}^{*}}{x_{2}^{*}}V_{2}\left( x^{*} \right) .$$

Therefore

$$W_{a}\left( x^{*} \right)=\frac{{2V}_{1}\left( x^{*} \right)+V_{2}\left( x^{*} \right)}{N\left( 2x_{1}^{*}+x_{2}^{*} \right)}=\frac{V_{2}\left( x^{*} \right)}{Nx_{2}^{*}}=\frac{V_{2}\left( x^{*} \right)+{2V}_{3}\left( x^{*} \right)}{N\left( x_{2}^{*}+2x_{3}^{*} \right)}=W_{A}\left( x^{*} \right). ∎$$

References

1. Hofbauer J, Sigmund K. Evolutionary games and population dynamics. Cambridge: Cambridge University Press; 1998. ISBN-13: 978-0521625708.2.

2. Haigh J. Game theory and evolution. Adv Appl Probab. 1975;7: 8-11. doi: 10.2307/1425844.

3. Hirsch MW, Smale S, Devaney RL Differential Equations, Dynamical Systems, and an Introduction to Chaos. 2nd ed. San Diego: Elsevier Academic Press; 2004.

4. Kong Q. A short course in ordinary differential equations. Springer; 2014. doi: 10.1007/978-3-319-11239-8.

5. Garay J, Garay BM, Varga Z, Csiszár V, Móri TF. To save or not to save your family member’s life? Evolutionary stability of self-sacrificing life history strategy in monogamous sexual populations. BMC Evol Biol. 2019;19: 147. doi: 10.1186/s12862-019-1478-0.
