# Supplementary material for: Matrix games between full siblings in Mendelian populations
Source: PLoS One. 2025 Sep 10;20(9):e0331044. doi: 10.1371/journal.pone.0331044 (PMC12422505; doi:10.1371/journal.pone.0331044)
Supplement: Attachment — Submitted filename: PLOS ONE - 2024 - Garay Matrix Mendel.pdf [file pone.0331044.s002.pdf]

Matrix game between full siblings in Mendelian populations

By József Garay, Tamás Varga , Villő Csiszár, Tamás F. Móri, and András Szilágyi

I like this paper a lot. When we try to explain kin selection or Hamilton's rule to students or to people outside academia, or when discuss it with each other, the go-to example is always full siblings – and for good reasons. Everyone in our profession has been doing this for about 60 years, and yet nobody has made a nuts-and-bolts, dynamic version of a model of a matrix game between full siblings. The authors however did exactly that, allowing for dominance and for deviations from equal gains from switching, and it is kind of unbelievable that they are the first. Whenever you think "why didn't anyone do this before?" that tends to be a symptom that they did something that is very welcome to the field.

I have a bunch of points regarding the presentation (which I think is not very good) but it should be clear that I like what they do, and that they do it well. The reason why I would like to emphasize that, has to do with the following.

The field of inclusive fitness can be rather toxic and has some unscientific characteristics. If this paper falls in the hands of these people, I expect that their review reports will claim that this paper is fundamentally flawed (without a real indication why), that this paper is doing the same as they have already done in some other paper (without indicating exactly how), and preferably both (without seeing the irony in the combination). This is then typically reason enough for editors to reject the paper, even though both these claims might be completely untrue (but hard to check for the editor). So, I want it to be known that the authors did something sound and solid.

That does not mean that the paper cannot be improved. It can, but that concerns the presentation rather than there being something incorrect or conceptually wrong about their results. The paper does not really illustrate very well what we can learn from the replicator dynamics-like technique they developed (while I think it is great that they did). The paper covers what the authors call "the alternating case", which adds to the overdose of cases and simplexes, and should really be an appendix, or maybe even a different paper. And the paper shies away a little bit from discussing how this relates to Hamilton's rule in much detail. Given the state of the field, I do understand why they would avoid too much reflection on how Hamilton's rule features in all of this, but given that we tend to use this example when we explain Hamilton's rule, it would be better if the authors did do this.

I was planning to give a very detailed description of how the presentation could be improved, and I am more than willing to do that still, but right now the deadline is approaching and I am running out of time. If the editors want to invite a resubmission, which I strongly suggest that they do, I am more than happy to make detailed suggestions how to improve the presentation of the paper, so that it makes it easy for the readers to understand why this is such a great contribution.
