# Supplementary material for: Matrix games between full siblings in Mendelian populations
Source: PLoS One. 2025 Sep 10;20(9):e0331044. doi: 10.1371/journal.pone.0331044 (PMC12422505; doi:10.1371/journal.pone.0331044)
Supplement: Attachment — Submitted filename: Response_to_Reviewers_GARAY_et_al_PlosONE.pdf [file pone.0331044.s003.pdf]

General response to the rewiers

We are grateful to the reviewers for their thorough evaluation and helpful suggestions. We have taken each of the comments into account, incorporating changes as recommended to improve the manuscript.

Below, our responses to each of the comments are highlighted in red. (The reviewers' comments are in black text.)

Along with the revised manuscript, we have also attached a marked-up version with the changes highlighted (old text is crossed out and highlighted in red, new text in cyan).

We note here that, due to technical reasons, we replaced Tables 5, 6, 8, 9, 11, 12, 14, and 15 with figures. The mentioned tables and the previous figure now correspond to the following figures: Tab. 5 **=>** Fig. 1, Tab. 6 **=>** Fig. 2, Tab. 8 **=>** Fig. 4, Tab. 9 **=>** Fig. 5, Tab. 11 **=>** Fig 6, Tab. 12 **=>** Fig. 7, Tab. 14 **=>** Fig. 10, and Tab. 15. **=>** Fig. 11.

As a result, the numbering of the remaining tables and original figures have also been adjusted: Tab. 7 **=>** Tab. 5, Tab. 10 **=>** Tab. 6, Tab. 13 **=>** Tab. 7, Fig. 1 **=>** Fig 3, Fig. 2 **=>** Fig 8, Fig. 3 **=>** Fig. 9.

We have expanded the previous reference list with 5 additional entries:

16. Allen B, McAvoy A. A mathematical formalism for natural selection with arbitrary spatial and genetic structure. J Math Biol. 2019;78: 1147–1210. doi: 10.1007/s00285-018-1305-z.
17. McAvoy A, Allen B. Fixation probabilities in evolutionary dynamics under weak selection. J Math Biol. 2021;82: 14. doi: 10.1007/s00285-021-01568-4.
18 Allen B, Khwaja AR, Donahue JL, Kelly TJ, Hyacinthe SR, Proulx J, et al. Nonlinear social evolution and the emergence of collective action. PNAS Nexus 2024;3(4): pgae131. doi: 10.1093/pnasnexus/pgae131.
19. Allen B, McAvoy A. The coalescent in finite populations with arbitrary, fixed structure. Theor Pop Biol. 2024;158: 150-169. doi: 10.1016/j.tpb.2024.06.004.
36. Santos FC, Pacheco JM, Lenaerts T. Evolutionary dynamics of social dilemmas in structured heterogeneous populations. PNAS Nexus. 2006;103: 3490-3494. doi: 10.1073/pnas.0508201103.

Response to Reviewer #1:

Thank youe for the thoughtful and constructive feedback, as well as for the valuable suggestions provided. We have carefully considered each point and have addressed these in the revised manuscript accordingly.

This paper develops a model of genotype dynamics, which combines Mendelian inheritance, replicator dynamics, and familial interactions. The model considers one autosomal locus with two alleles and thus three genotypes, which are mapped to phenotypes. There are then interactions between siblings that may be competitive or cooperative. These are represented by a 2x2 symmetric game where the phenotypes are strategies (either pure or mixed). Interactions occur between siblings, which creates a type of group selection dynamic. The paper develops a replicator-like equation for this system and presents the stability conditions (definitions and conditions) for this model, which are analogous to those in evolutionary game theory. In particular, it explores the conditions for stability of homozygous states and analyzes the scenario where the symmetric game is a Prisoner's Dilemma. Several parsimonious conditions are found under which collaboration/cooperation is supported. Numerical solutions are used for more complicated scenarios. The paper is well written and thorough, and the model is interesting with many possible extensions as it folds genotypes and familial interactions into evolutionary game theory in a compelling and novel way. The results are well founded, and I found no errors in the calculations (the SI provides sufficient further details on calculations). In general, I enjoyed reading this paper and support its publication. I have only a few minor comments and questions detailed below.

Table 1 caption: Please remind the reader what n is.

We have made this correction everywhere it was missing (see Tab. 1, Tab. 4, Tab. 5 (old Tab. 7)).

Is there any intuition about the conditions 10 and 11?

These conditions were obtained by analytic considerations. Unfortunately, we cannot support them intuitively; inequality (11) seems particularly difficult.

And, how do they relate to 2x2 games such as the hawk-dove, stag hunt, prisoner's dilemma, and stag hunt? One may represent the qualitative space of 2x2 games in 2-dimensional space with parameters S and T (for an example see Santos 2006). The four quadrants are thus each a different game. It might be illustrative to represent the regions where the conditions 10 and 11 are met/not met on this plane. (Santos, Francisco C., Jorge M. Pacheco, and Tom Lenaerts. "Evolutionary dynamics of social dilemmas in structured heterogeneous populations." Proceedings of the National Academy of Sciences 103.9 (2006): 3490-3494.)

Based on our current results, we believe that such a classification would be quite an extensive task, likely requiring a separate manuscript(s), so we have decided not to undertake it at this time. In the classical evolutionary matrix games, in the four types of games, there are either a pure globally stable ESS, or a globally stable mixed ESS or two pure stable ESS (bistablity). In our case, the most difficult mathematical issue is the existence of global stability. Furthermore, we even do not know if it is possible, and if so, under what conditions there will be a cyclic solution of the genotype dynamics. As a first step, we plan to investigate the case where the conflict between siblings is determined by the hawk-dove setup. We think that many cases are possible in this model as well, similar to the PD game under investigation.

The proposed paper has been referred to, see line 676 (p 26) in the revised manuscript:

line 676 “*We note that standard models of group selection focus primarily on asexual populations (e.g. [35-36], thus …*”

Tables 2 and 3: By "suspected" is it meant that a necessary but not sufficient condition is met?

You are right. The results in Tables 2 and 3 are based on our earlier results presented in [3]. There we gave first and second order conditions (inequalities) for a genotype to be evolutionarily stable in the model examined there. If in the first order condition the inequality holds in the opposite direction, the corresponding vertex becomes a repellor. This is not necessarily so if the inequality in a second order condition is reversed, because in this way we only obtain a condition that is necessary but not sufficient for getting a repellor. As the inequalities in question in Table 3 were obtained from second order conditions, they make it likely, though not prove, that the vertex under consideration is a repellor.

Alternative states on Pg, 16 Line 407: Would not both defect in this case? If not, why not?

Our classification simply indicates that if ASEXUAL PLAYERS were aiming to maximize their joint survival, one would need to defect while the other cooperates. Whether this actually occurs under natural selection is one of the key questions we examine in this paper. Table 15 provides an overview of the main possible cases. Here as well, no intuitive answer can be given as to why the defect homozygote is not always globally stable. (A similar explanation has been added to the manuscript following the introduction to these cases.)

Response to Reviewer #2

Thank you very much for your supportive feedback on our manuscript. We truly appreciate your positive evaluation, and we would be more than happy to receive any suggestions you may have for further improvements.

In any case, we note that we investigate a population genetic model with mathematical rigor. Our direct goal is not to examine Hamilton's original rule. The main reason for this is that Hamilton considered a "viscous" population, while we consider a population of monogamous families. Thus, it is not an obvious question how our results compare to Hamilton's original result. But it is obvious that Haldane's familial selection is a special case of the general kin selection, thus the general kin selection theory must give back our reults.

I like this paper a lot. When we try to explain kin selection or Hamilton's rule to students or to people outside academia, or when discuss it with each other, the go-to example is always full siblings – and for good reasons. Everyone in our profession has been doing this for about 60 years, and yet nobody has made a nuts-and-bolts, dynamic version of a model of a matrix game between full siblings. The authors however did exactly that, allowing for dominance and for deviations from equal gains from switching, and it is kind of unbelievable that they are the first. Whenever you think "why didn't anyone do this before?" that tends to be a symptom that they did something that is very welcome to the field.

I have a bunch of points regarding the presentation (which I think is not very good) but it should be clear that I like what they do, and that they do it well. The reason why I would like to emphasize that, has to do with the following.

The field of inclusive fitness can be rather toxic and has some unscientific characteristics. If this paper falls in the hands of these people, I expect that their review reports will claim that this paper is fundamentally flawed (without a real indication why), that this paper is doing the same as they have already done in some other paper (without indicating exactly how), and preferably both (without seeing the irony in the combination). This is then typically reason enough for editors to reject the paper, even though both these claims might be completely untrue (but hard to check for the editor). So, I want it to be known that the authors did something sound and solid.

That does not mean that the paper cannot be improved. It can, but that concerns the presentation rather than there being something incorrect or conceptually wrong about their results. The paper does not really illustrate very well what we can learn from the replicator dynamics-like technique they developed (while I think it is great that they did). The paper covers what the authors call "the alternating case", which adds to the overdose of cases and simplexes, and should really be an appendix, or maybe even a different paper. And the paper shies away a little bit from discussing how this relates to Hamilton's rule in much detail. Given the state of the field, I do understand why they would avoid too much reflection on how Hamilton's rule features in all of this, but given that we tend to use this example when we explain Hamilton's rule, it would be better if the authors did do this.

I was planning to give a detailed description of how the presentation could be improved, and I am more than willing to do that still, but right now the deadline is approaching and I am running out of time. If the editors want to invite a resubmission, which I strongly suggest that they do, I am more than happy to make detailed suggestions how to improve the presentation of the paper, so that it makes it easy for the readers to understand why this is such a great contribution.
