# Supplementary material for: Matrix games between full siblings in Mendelian populations
Source: PLoS One. 2025 Sep 10;20(9):e0331044. doi: 10.1371/journal.pone.0331044 (PMC12422505; doi:10.1371/journal.pone.0331044)
Supplement: Attachment — Submitted filename: PLOS ONE - 2024 - Garay Matrix Mendel R1.pdf [file pone.0331044.s004.pdf]

Matrix game between full siblings in Mendelian populations - R1

By József Garay, Tamás Varga , Villő Csiszár, Tamás F. Móri, and András Szilágyi

Before I make a few broad suggestions how to improve the presentation, I want to emphasize how much I like the paper. This I would like to do to make sure that the authors understand that the critical remarks about the presentation are out of appreciation of what is in the paper, and that I am motivated by the wish that the paper is read, and the results are appreciated for what they are – and not disregarded or overlooked.

So, again, I would think that one of the selling points of this paper is that it does what should have been done long before, and that is ***not*** to take the shortcut of modeling interactions between siblings with an asexual model of kin selection; allow for relatedness to be $r = \frac{1}{2}$; and then think as interactions between siblings. Instead, this paper (also building on previous work by the same authors) does the homework right, and makes a proper model with sexual reproduction, with siblings that do get their genes from a father and a mother. Part of the improved presentation could, or should, be to stress this simple fact early on; point to the lack of a paper that models our prime example for kin selection properly, point to a few papers that take the asexual shortcut, and point out that this paper does not do that, with interesting differences. (Many papers also may not aim at doing what this paper does, but it is remarkable that we have a million papers for which asexual reproduction is the most natural interpretation, while everyone uses the sibling example to explain kin selection).

It seems that the paper focuses on identifying when we can expect one or the other behaviour to go to fixation; when we can expect bistability; and when we can expect coexistence. I think that this is a very good point to focus on, also if the paper wants to stress what having a proper model with sexual (rather than asexual) reproduction adds to our understanding of the dynamics. For that, it might help to stress a bit more that there are two relevant layers in the model; the payoff function and the genotype-phenotype mapping, both of which one could imagine contributing to this. This suggests that an interesting question to give a precise answer to here would be which of these two determines whether there is bistability or coexistence, or, more precisely, for which combinations of the two layers we get bistability or coexistence.

The introduction and abstract are a bit unspecific about this and they contain information that distracts from the main quest. The first sentence of the abstract, for instance, is way too opaque and technical, and only understandable by readers that are already familiar with the setup of the paper – which the authors obviously are, but most readers are not.

What also does not help is that the paper considers two scenarios. The first is called “cooperation” (collaborating case). The second is called “defector-cooperator strategy pair”

(alternating case). First of all this is weird terminology, which I also do not really recognize from elsewhere. I would think "collaborating" is just a synomym for cooperation, and unless this is established terminology in some corner of the literature, this does not clarify anything. "Alternating" on the other hand is what a pair could do, in case it would repeatedly interact and have this payoff matrix, and the pair would want to get the largest combined fitnesses, and also distribute those in a balanced way. None of that happens in the paper, though, so why the word "alternating" is used, is unclear. (Also, this is a place where articles should be used; ***the*** collaborating/alternating case)

But, what is more important than the hurdle of the unusual and ambiguous terminology, is that for the second case it is super-obvious that neither can go to fixation. Therefore this case is an unneccessary distraction, especially given that, as of yet, in the introduction and abstract, it is not clear what makes bistability or coexistence possible in what the authors call the "collaborating" case. So I would suggest just to focus on the first case, with more precision, and let the second case be discussed in the appendix – which also allows the paper to not waste the readers attention in the abstract and intro on trying to figure out what these two cases are (most people will think of the first case when they hear the word prisoner's dilemma anyway). Again, because I have seen the paper before, I know which payoff matrices go with the "collaborating" and the "alternating" case, but at this point in the abstract, the unsuspecting reader can only guess, on the basis of a description that the authors surely think should be enough, but that really is not enough for readers to figure out what sets these two cases apart. Really explaining it there, on the other hand, would take too much time and space away from more interesting things that it could zero in on.

I do understand the wish for generality, and therefore the wish to cover all prisoners dilemmas. The wish to cover everything would be an argument for including the "alternating" case. However, it is also true that even if the "alternating" case is included, as it currently is, the paper still draws a line somewhere. It looks at prisoner's dilemmas, and not at matrices for games that are not prisoner's dilemma, that one could also study within the same framework. Therefore, I would think that one would have to ask what the added value is of drawing the line here (for the main text, and the main focus), where it includes the "alternating" case. I see more downsides than upsides of that. So, to be short, I would put the "alternating" case at in the appendix, to satisfy the desire for (more) completeness, but not have this as a hurdle to understanding how, for instance, prisoners' dilemmas with or without equal gains from switching lead to different results, and how that interacts with the genotype-phenotype mapping. And, again, the terminology here should be improved.

I also want to respond to the author's response to my earlier report. The authors write:

In any case, we note that we investigate a population genetic model with mathematical rigor. Our direct goal is not to examine Hamilton's original rule. The main reason for this is that Hamilton considered a "viscous" population, while we consider a population of

monogamous families. Thus, it is not an obvious question how our results compare to Hamilton's original result.

In response to this, I would like to say two things. The first is that this contains an incorrect claim. A “viscous” poipulation is only one of the possible sources of relatedness suggested by Hamilton. As a matter of fact, this is an option that actually turned out to not work, or at least requires a deeper understanding of how kin selection works, if it does, to select altruistic/cooperative traits. Wilson, Dugatkin an Pollock (1992), and, subsequently, Taylor (1992), discovered the cancellation effect, when they were asking theirselves how viscosity could produce cooperation.

However, Hamilton (1964) does not model viscosity, or any other source for relatedness. Hamilton (1964) just ***postulated*** a vector of fitness effects, without specifying where this comes from.

Moreover, given that all of us use the example of siblings when explaining kin selection and Hamilton’s rule, I think it is important to relate this, not just to Hamilton’s original paper (however loosely), but also, and maybe more importantly so, to later ways in which Hamilton’s rule has been re-derived. This would also include models that one would naturally interpret as models with asexual reproduction, like Taylor (1989), to just give one example.

I am aware that this requires reorganizing and reworking the paper, but I think it will really pay off as for the dissemination of the results in this paper.

Smaller details

Throughout, articles are missing (for example; in donation game → in the/a donation game, but also in the title, where maybe the plural). Also, the donation game in this setting is really a prisoners dilemma with equal gains from switching, as both siblings can choose.

Line 133: Is $nk(ij)$ best described as a parameter? Later, it seems to be a function of the population state x.

References

Taylor, Peter D. "Evolutionary stability in one-parameter models under weak selection." *Theoretical population biology* 36.2 (1989): 125-143.

Taylor, Peter D. "Altruism in viscous populations—an inclusive fitness model." *Evolutionary ecology* 6 (1992): 352-356.

Wilson, David Sloan, Gregory B. Pollock, and Lee A. Dugatkin. "Can altruism evolve in purely viscous populations?." *Evolutionary ecology* 6 (1992): 331-341.
