# Supplementary material for: Matrix games between full siblings in Mendelian populations
Source: PLoS One. 2025 Sep 10;20(9):e0331044. doi: 10.1371/journal.pone.0331044 (PMC12422505; doi:10.1371/journal.pone.0331044)
Supplement: Attachment — Submitted filename: Response_2_to_Reviewers_GARAY_et_al_PlosONE.pdf [file pone.0331044.s005.pdf]

General response #2 to the rewiers

We appreciate the reviewers' efforts and valuable advice.

Below, our responses to each of the comments are highlighted in red. (The reviewers' comments are in black text.)

The changes can be summarized as follows:

Several references have been added that discuss interactions between siblings within either a sexual haploid or an asexual model framework. These two approaches are quite common. As a complement to these, the model presented in this manuscript focuses on diploidy, highlighting the role of the phenotype-(diploid) genotype mapping in evolutionary dynamics as one of its key findings.

The previous terms "collaborating" case have been replaced by "cooperation" case and we more often use a descriptive explanations for the cooperation and alternating cases. Hopefully, this will make it easier for readers to navigate the terminology, especially for those who do not wish to delve into the details of the paper or are only glancing at specific sections.

Along with the revised manuscript, we have also attached a marked-up version with the changes highlighted (old text is crossed out and highlighted in red, new text in cyan).

Response to Reviewer #1:

We appreciate the previous comments and helpful suggestions. In response to the other reviewer's recommendations, we have made a few additional changes and added a couple of paragraphs to the Introduction and Conclusion.

Response to Reviewer #2

We are grateful for the comments, helpful remarks, and suggestions. Taking these into account, we have made efforts to revise and improve the previous version of the manuscript.

[REVIEWER] Before I make a few broad suggestions how to improve the presentation, I want to emphasize how much I like the paper. This I would like to do to make sure that the authors understand that the critical remarks about the presentation are out of appreciation of what is in the paper, and that I am motivated by the wish that the paper is read, and the results are appreciated for what they are – and not disregarded or overlooked.

[REVIEWER] So, again, I would think that one of the selling points of this paper is that it does what should have been done long before, and that is not to take the shortcut of modeling interactions between siblings with an asexual model of kin selection; allow for relatedness to be r=1/2; and then think as interactions between siblings. Instead, this paper (also building on previous work by the same authors) does the homework right, and makes a proper model with sexual reproduction, with siblings that do get their genes from a father and a mother. Part of the improved presentation could, or should, be to stress this simple fact early on; point to the lack of a paper that models our prime example for kin selection properly, point to a few papers that take the asexual shortcut, and point out that this paper does not do that, with interesting differences. (Many papers also may not aim at doing what this paper does, but it is remarkable that we have a million papers for which asexual reproduction is the most natural interpretation, while everyone uses the sibling example to explain kin selection).

[AUTHORS] As mentiond, in the previous review, "The field of inclusive fitness can be rather toxic and has some unscientific characteristics." Our previous experiences support this opinion. Therefore, whenever possible, we strictly focus on our population genetic model. If we start arguing in the *Introduction* in defense of our model—explaining why genotype-centered models are necessary for studying kin selection in diploid populations—we fear that we may lose some potential readers, particularly those who strongly adhere to Hamilton's rule. We must mention that the Editors of *BMC Ecol Evol* have already forced us to formulate similar arguments (see [59] in the references).

[AUTHORS] Accordingly, we have tried to phrase our statements cautiously. We have rewritten and expanded the second paragraph of *Introduction* (see lines 65-73) and added three paragraphs to the end of *Conclusion* (see lines 815-844), and expanded the reference list (from [5] to [19] and [59]).

[REVIEWER] It seems that the paper focuses on identifying when we can expect one or the other behaviour to go to fixation; when we can expect bistability; and when we can expect coexistence. I think that this is a very good point to focus on, also if the paper wants to stress what having a proper model with sexual (rather than asexual) reproduction adds to our understanding of the dynamics. For that, it might help to stress a bit more that there are two relevant layers in the model; the payoff function and the genotype-phenotype mapping, both of which one could imagine contributing to this. This suggests that an interesting question to give a precise answer to here would be which of these two determines whether there is bistability or coexistence, or, more precisely, for which combinations of the two layers we get bistability or coexistence.

[AUTHORS] Following the suggestion, we have inserted a sentence into the last paragraph of *Introduction* (the last but one sentence) to express our intention to examine the combined effect of the payoff matrix and the genotype-phenotype mapping on the evolutionary outcome (see lines 112-114).

[REVIEWER] The introduction and abstract are a bit unspecific about this and they contain information that distracts from the main quest. The first sentence of the abstract, for instance, is way too opaque and technical, and only understandable by readers that are already familiar with the setup of the paper – which the authors obviously are, but most readers are not.

[AUTHORS] The beginning of the abstract has been rephrased. We believe that the first two sentences of the abstract summarizes our most important result from a biomathematical perspective (Theorem 1 on p 9), as it holds even for arbitrary interactions within a family (see [59]). In this paper, we focus on matrix games and the prisoner's dilemma only as an application.

[REVIEWER] What also does not help is that the paper considers two scenarios. The first is called "cooperation" (collaborating case). The second is called "defector-cooperator strategy pair" (alternating case). First of all this is weird terminology, which I also do not really recognize from elsewhere. I would think "collaborating" is just a synomym for cooperation, and unless this is established terminology in some corner of the literature, this does not clarify anything. "Alternating" on the other hand is what a pair could do, in case it would repeatedly interact and have this payoff matrix, and the pair would want to get the largest combined fitnesses, and also distribute those in a balanced way. None of that happens in the paper, though, so why the word "alternating" is used, is unclear. (Also, this is a place where articles should be used; the collaborating/alternating case)

[AUTHORS] We have reworded the relevant parts of the Abstract and the Author Summary. The term ``collaborating case" was replaced by ``cooperation case''. We retained the term "alternating case" However, we supplemented the text in several places with descriptive explanations to ensure that readers who only glance at certain parts of the manuscript can also understand the meaning of the cases.

[REVIEWER] But, what is more important than the hurdle of the unusual and ambiguous terminology, is that for the second case it is super-obvious that neither can go to fixation. Therefore this case is an unneccessary distraction, especially given that, as of yet, in the introduction and abstract, it is not clear what makes bistability or coexistence possible in what the authors call the "collaborating" case. So I would suggest just to focus on the first case, with more precision, and let the second case be discussed in the appendix – which also allows the paper to not waste the readers attention in the abstract and intro on trying to figure out what these two cases are (most people will think of the first case when they hear the word prisoner's dilemma anyway). Again, because I have seen the paper before, I know which payoff matrices go with the "collaborating" and the "alternating" case, but at this point in the abstract, the unsuspecting reader can only guess, on the basis of a description that the authors surely think should be enough, but that really is not enough for readers to figure out what sets these two cases apart. Really explaining it there, on the other hand, would take too much time and space away from more interesting things that it could zero in on.

[REVIEWER] I do understand the wish for generality, and therefore the wish to cover all prisoners dilemmas. The wish to cover everything would be an argument for including the "alternating" case. However, it is also true that even if the "alternating" case is included, as it currently is, the paper still draws a line somewhere. It looks at prisoner's dilemmas, and not at matrices for games that are not prisoner's dilemma, that one could also study within the same framework. Therefore, I would think that one would have to ask what the added value is of drawing the line here (for the main text, and the main focus), where it includes the "alternating" case. I see more downsides than upsides of that. So, to be short, I would put the "alternating" case at in the appendix, to satisfy the desire for (more) completeness, but not have this as a hurdle to understanding how, for instance, prisoners' dilemmas with or without equal gains from switching lead to different results, and how that interacts with the genotype-phenotype mapping. And, again, the terminology here should be improved.

[AUTHORS] It may be expected that natural selection favors the cooperator-defector pair and thus a mixed population of cooperators and defectors if pure cooperation does not maximize the combined survival of the two interacting siblings. However, it is not self-evident that this will always be the case under different genotype-phenotype mappings. This was one of the main reasons for considering diploid genotype frequencies and the genotype-phenotype mapping.

[AUTHORS] Indeed, as the obtained results show, the situation is not always straightforward, at least in terms of the stability of the genotype dynamics, even when the cooperator-defector strategy pair maximizes the siblings' survival probability in a prisoner's dilemma game. For example, in the case of the A_{10} payoff matrix (in Fig 11), when inheritance is intermediate, a population consisting solely of defectors is the only stable state. However, if the cooperative trait is inherited recessively or dominantly, a mixed population of cooperators and defectors turns out to be stable. This seems difficult to explain intuitively.

[AUTHORS] Similarly, only after performing the calculations can we confidently state that a purely cooperative population cannot become fixed when the cooperator-defector strategy pair maximizes the total payoff of two interacting siblings.

[AUTHORS] Therefore, we believe that our results for this case should definitely be included in the main text.

[REVIEWER] I also want to respond to the author's response to my earlier report. The authors write:

[REVIEWER] In any case, we note that we investigate a population genetic model with mathematical rigor. Our direct goal is not to examine Hamilton's original rule. The main reason for this is that Hamilton considered a "viscous" population, while we consider a population of monogamous families. Thus, it is not an obvious question how our results compare to Hamilton's original result.

[REVIEWER] In response to this, I would like to say two things. The first is that this contains an incorrect claim. A "viscous" poipulation is only one of the possible sources of relatedness suggested by Hamilton. As a matter of fact, this is an option that actually turned out to not work, or at least requires a deeper understanding of how kin selection works, if it does, to select altruistic/cooperative traits. Wilson, Dugatkin an Pollock (1992), and, subsequently, Taylor (1992), discovered the cancellation effect, when they were asking theirselves how viscosity could produce cooperation.

[REVIEWER] However, Hamilton (1964) does not model viscosity, or any other source for relatedness. Hamilton (1964) just postulated a vector of fitness effects, without specifying where this comes from. Moreover, given that all of us use the example of siblings when explaining kin selection and Hamilton's rule, I think it is important to relate this, not just to Hamilton's original paper (however loosely), but also, and maybe more importantly so, to later ways in which Hamilton's rule has been re-derived. This would also include models that one would naturally interpret as models with asexual reproduction, like Taylor (1989), to just give one example.

[REVIEWER] I am aware that this requires reorganizing and reworking the paper, but I think it will really pay off as for the dissemination of the results in this paper.

[AUTHORS] Based on the response above, we have inserted three additional paragraphs at the end of *Conclusion* (see lines 815-844), and we also address this point in the revised second paragraph of

Introduction (see lines 65-73) . Thank you for drawing our attention to several articles, which allowed us to expand our references.

[REVIEWER] Smaller details

[REVIEWER] Throughout, articles are missing (for example; in donation game --> in the/a donation game, but also in the title, where maybe the plural). Also, the donation game in this setting is really a prisoners dilemma with equal gains from switching, as both siblings can choose.

[AUTHORS] Thank you for the comment; we have made an effort to improve the use of articles throughout the manuscript.

[REVIEWER] Line 133: Is nk(ij) best described as a parameter? Later, it seems to be a function of the population state x.

[AUTHORS] In general, $n_{k(ij)}$ may also depend on the composition *x* of the population. Since this does not introduce significant complications in the general formulation of the genotype dynamics, we initially assumed its dependence on *x* (see the displayed formula in line 163). However, in the subsequent applications (from section titled by ``Application: Filial selection with a matrix game…”), $n_{k(ij)}$ is considered independent of *x* and depends only on the parents' genotypes and the matrix game. This simplification makes the calculations more straightforward and ensures that the effect of the population composition does not obscure the role of genotype-phenotype mapping and the matrix game in the evolutionary outcomes. To clarify this point, we have supplemented Remark 2 on pages 6-7.

[REVIEWER] References

Taylor, Peter D. "Evolutionary stability in one-parameter models under weak selection." Theoretical population biology 36.2 (1989): 125-143.

Taylor, Peter D. "Altruism in viscous populations—an inclusive fitness model." Evolutionary ecology 6 (1992): 352-356.

van Veelen, M., Allen, B., Hoffman, M., Simon, B., & Veller, C. (2017). Hamilton's rule. Journal of Theoretical Biology, 414, 176-230.

Wilson, David Sloan, Gregory B. Pollock, and Lee A. Dugatkin. "Can altruism evolve in purely viscous populations?." Evolutionary ecology 6 (1992): 331-341.
