# Supplementary material for: Matrix games between full siblings in Mendelian populations
Source: PLoS One. 2025 Sep 10;20(9):e0331044. doi: 10.1371/journal.pone.0331044 (PMC12422505; doi:10.1371/journal.pone.0331044)
Supplement: Attachment — Submitted filename: PLOS ONE - 2024 - Garay Matrix Mendel R.pdf [file pone.0331044.s006.pdf]

Matrix game between full siblings in Mendelian populations - R1

By József Garay, Tamás Varga , Villő Csiszár, Tamás F. Móri, and András Szilágyi

I have expressed my appreciation for this paper, but I also find it a bit frustrating that the authors only pick up on suggestions very minimally, and incrementally. I find that frustrating, because I really want this paper to be published, but I also want people to be able to read it, understand it, and take it seriously. The authors also do not seem to be aware of their limitations in the English language and in writing in general. This puts me in a difficult situation; it is not my job to rewrite their paper, but the authors seem incapable or unwilling to present their results better.

To indicate this, let me just go over the title and abstract in some detail, as a sample.

Title: Matrix game between full siblings in Mendelian populations

This is not correct English. Correct options in are "*Matrix game**s** between full siblings in Mendelian populations*" or "***A** matrix game between full siblings in Mendelian populations*" or "***The** matrix game between full siblings in Mendelian populations*". To have a title in bad English is just embarrassing, and it should not be the case that the title alone already gives readers an excuse to ignore the paper. Out of the three correct options, the first would be the best, because the paper allows for a variety of 2x2 payoff matrices.

Abstract: The genotype dynamics pertain to Mendelian populations in which diploid individuals mate.

This is needlessly ambiguous. Which genotype dynamcs are we talking about here? Do you introduce them in this paper? Why not something like: we formulate/present/consider genotype dynamics in Medelian …?

It describes the temporal changes in the frequencies of the possible genotypes within a population of individuals following Mendelian inheritance rules.

This is an otherwise OK sentence, but why the "temporal"? Most changes are changes over time. This makes readers think they may have missed something.

We show that static evolutionary stability implies the stability of the corresponding interior equilibrium point in the genotype dynamics.

The static evolutionary stability is something that is also defined in this paper, right? Or is it an established definition?

We apply our findings to familial selection in a diploid, panmictic population, where the survival rates of siblings within monogamous and exogamous families are determined by a

matrix game, and the behavior is uniquely determined by an autosomal recessive-dominant or intermediate allele pair. We provide conditions for the existence of each homozygote.

What do you mean “conditions for the existence of each homozygote”? Do you mean “conditions under which each homozygote is stable?”

In our numerical investigations of the prisoner’s dilemma between siblings, we distinguish between two cases depending on whether the sum of the survival rates of siblings involved in the interaction is greater in cooperator-cooperator interactions (cooperation case) or in cooperator-defector interactions (alternating case).

What are “numerical investigations”? You mean that you choose values for parameters and draw the dynamics on the simplex? Numerical sounds like it could be an alternative to analytic, which is not what any of this is about. Also, as before, I don’t like the terms “cooperation case” and “alternating case”. Whether or not to alternate is a choice one could make, not a property of a payoff matrix.

Based on the stability of the pure cooperator and defector states, we provide a potential classification of the genotype dynamics. We find that the pure cooperator population cannot fixate in the alternating case.

What does it mean for a cooperator population to fixate? You mean a mutant cooperator to go to fixation? Also, as I said before, this observation is trivial.

However, in the cooperation case, fixation is possible but not necessary, since bistability, coexistence, …

This is not a sentence. What does this mean? Totally vague.

… moreover, the monostable fixation of the pure defector state can also occur due to the interplay between the phenotypic payoff function and the genotype-phenotype mapping, which collectively determine the outcome of natural selection.

What does this mean? We are doing infinite population, deterministic dynamics, so why use the word fixation? Why not use terms like stable monomorphic state or something like that. And why leave the interplay a black box? These two ingredients are not both needed in conjunction to get bistability and/or coexistence.

In the donation game, the classical Hamilton’s rule implies the fixation of the cooperation in all considered genotype-phenotype mappings.

First of all “the cooperation” should be “cooperation”. Also, this is a strange way to put this. Hamilton’s rule does not imply fixation of cooperation in general, it is a rule that describes when cooperation does and when it does not fixate (again, if that is the teminology used, which I would argue against, because it really staes when cooperation is selected for, and with frequency dependent costs and/or benefits that can allow for stable mixtures).

And these are just superficial comments about the language. The abstract also does not really focus on what is most important.

I will leave it at this, but the rest of the paper has similar suboptimalities in the language.

Just to comment on something that the authors say in their response, in defense of not clarifying the usefulness of their approach in relation tot the inclusive fitness literature:

As mentiond, in the previous review, “The field of inclusive fitness can be rather toxic and has some unscientific characteristics.” Our previous experiences support this opinion. Therefore, whenever possible, we strictly focus on our population genetic model. If we start arguing in the Introduction in defense of our model—explaining why genotype-centered models are necessary for studying kin selection in diploid populations—we fear that we may lose some potential readers, particularly those who strongly adhere to Hamilton’s rule.

I do of course totally sympathise, but I think the risk-assesment by the authors is wrong. There are two aspects: 1) getting a paper published and 2) getting people to read it. Regarding point 1) you have two sympathetic reviewers, so there is no need to be overly cautious. Regarding point 2) the IF people will not read your paper anyway. Therefore, you should explain to neutral, non-partisan readers why this is helpful, and those are typically not offended by clarity and precision.
