# Supplementary material for: Matrix games between full siblings in Mendelian populations
Source: PLoS One. 2025 Sep 10;20(9):e0331044. doi: 10.1371/journal.pone.0331044 (PMC12422505; doi:10.1371/journal.pone.0331044)
Supplement: Attachment — Submitted filename: Response_3_to_Reviewers_GARAY_et_al_PlosONE.pdf [file pone.0331044.s007.pdf]

# General response #3 to the rewiers

We sincerely appreciate the reviewers' time, effort, and constructive feedback.

Below, our responses to each of the comments are highlighted in red. (The reviewers' comments are in black text.)

The changes can be summarized as follows:

In the title, the word "game" has been replaced by its plural form "games".

We did not alter the structure or core content of the manuscript. However, we made an effort to improve the clarity, readability, and precision of the text. This affected most sentences, as can be followed in the marked-up version.

We replaced the terms *"cooperation case"* and *"alternating case"* with *"coordinated case"* and *"anti-coordinated case"*, respectively, by analogy with the terminology of coordination games (in which a player's payoff is higher when playing the same strategy as the opponent) and anti-coordination games (where a player's payoff is higher when playing a different strategy). Nevertheless, the concepts are not identical: in our setting, the relevant criterion is the maximization of the **joint** payoff of the two players, whereas in coordination and anti-coordination games the focus is on the **individual** payoffs. To clarify this distinction, we added reference [46].

We had previously used the terms "fixate" and "fixation" informally in several places as alternatives to "evolutionary stability" and, by implication through our model, to "asymptotic stability" in the context of genotype dynamics (since in our framework evolutionary stability implies asymptotic stability). However, these expressions may suggest a stochastic framework, which is not the case here. Therefore, where their meaning might be misleading, we revised the text and replaced them with "stable" or "stability," as appropriate.

In addition, we extended the list of references to include works [37]–[39], which also make use of mating tables.

Along with the revised manuscript, we have also attached a marked-up version with the changes highlighted (old text is crossed out and highlighted in red, new text in cyan).

## Response to Reviewer 1:

We sincerely appreciate your continued support and positive evaluation of our work. In this round of revision, we primarily focused on improving the clarity and precision of the text throughout the manuscript and made some adjustments to terminology and references. We are grateful for your kind recommendation for acceptance.

## Response to Reviewer 2:

[AUTHORS] We thank the reviewer for the thorough and constructive feedback. In response to the detailed comments and suggestions, we revised the manuscript with the aim of improving its clarity, precision, and overall presentation.

[REVIEWER] I have expressed my appreciation for this paper, but I also find it a bit frustrating that the authors only pick up on suggestions very minimally, and incrementally. I find that frustrating, because I really want this paper to be published, but I also want people to be able to read it, understand it, and take it seriously. The authors also do not seem to be aware of their limitations in the English language and in writing in general. This puts me in a difficult situation; it is not my job to rewrite their paper, but the authors seem incapable or unwilling to present their results better.

[AUTHORS] We thank the reviewer for the helpful linguistic suggestions regarding the abstract. We have incorporated these suggestions, and based on them, we have also made efforts to improve the overall language quality of the manuscript.

[REVIEWER] To indicate this, let me just go over the title and abstract in some detail, as a sample.

*Title: Matrix game between full siblings in Mendelian populations*

This is not correct English. Correct options in are “Matrix game**s** between full siblings in Mendelian populations” or “A matrix game between full siblings in Mendelian populations” or “The matrix game between full siblings in Mendelian populations”. To have a title in bad English is just embarrassing, and it should not be the case that the title alone already gives readers an excuse to ignore the paper. Out of the three correct options, the first would be the best, because the paper allows for a variety of 2x2 payoff matrices.

[AUTHORS] It is done.

[REVIEWER] Abstract: *The genotype dynamics pertain to Mendelian populations in which diploid individuals mate*.

This is needlessly ambiguous. Which genotype dynamcs are we talking about here? Do you introduce them in this paper? Why not something like: We formulate/present/consider genotype dynamics in Medelian …?

[AUTHORS] We have followed the suggestion and revised the sentence accordingly. We note that this genotype dynamics framework was previously introduced in the appendix of an earlier paper of ours, where it was applied only to populations consisting of pure homozygotes. Our aim there was to show that the ESGD (Evolutionarily Stable Genotype Distribution) definition introduced in that context also guarantees dynamic stability.

[REVIEWER] *It describes the temporal changes in the frequencies of the possible genotypes within a population of individuals following Mendelian inheritance rules.*

This is an otherwise OK sentence, but why the “temporal”? Most changes are changes over time. This makes readers think they may have missed something.

[AUTHORS] The word “temporal” was cancelled.

[REVIEWER] *We show that static evolutionary stability implies the stability of the corresponding interior equilibrium point in the genotype dynamics.*

The static evolutionary stability is something that is also defined in this paper, right? Or is it an established definition?

[AUTHORS] We thank the reviewer for raising this point. In our earlier work [3], we defined static evolutionary stability only for pure states, where the population consists entirely of one homozygote genotype. In the present paper, we introduce the corresponding definition for mixed states, where all three genotypes are present. We make this clear immediately before the formal definition of the mixed ESGD (see lines 200–208). To further clarify this in the abstract, we have expanded the sentence in question.

[REVIEWER] *We apply our findings to familial selection in a diploid, panmictic population, where the survival rates of siblings within monogamous and exogamous families are determined by a matrix game, and the behavior is uniquely determined by an autosomal recessive-dominant or intermediate allele pair. We provide conditions for the existence of each homozygote.*

What do you mean "*conditions for the existence of each homozygote*"? Do you mean "*conditions under which each homozygote is stable?*"

[AUTHORS] This is right — as originally phrased, the sentence is indeed unclear. What we meant was that we provide conditions for the evolutionary stability of the pure homozygote states. We have revised the sentence accordingly to clarify this.

[REVIEWER] *In our numerical investigations of the prisoner's dilemma between siblings, we distinguish between two cases depending on whether the sum of the survival rates of siblings involved in the interaction is greater in cooperator-cooperator interactions (cooperation case) or in cooperator-defector interactions (alternating case).*

What are "numerical investigations"? You mean that you choose values for parameters and draw the dynamics on the simplex? Numerical sounds like it could be an alternative to analytic, which is not what any of this is about. Also, as before, I don't like the terms "cooperation case" and "alternating case". Whether or not to alternate is a choice one could make, not a property of a payoff matrix.

[AUTHORS] It is right that the term "numerical investigations" is misleading in this context. We are not referring to numerical analysis in contrast to analytic results, but rather to illustrative parameter examples that demonstrate the possible types of payoff matrices arising from the stability conditions and genotype–phenotype mappings. We have rephrased the relevant text accordingly to avoid confusion.

Regarding the labels "cooperation case" and "alternating case," we agree that they were not ideal. We have replaced them with "coordinated case" and "anti-coordinated case," respectively. These terms have been adopted from Szabó et al., *Evolutionary potential games on lattices*. , Physics Reports, Volume 624, 2016, Pages 1-60, https://doi.org/10.1016/j.physrep.2016.02.006.

[REVIEWER] *Based on the stability of the pure cooperator and defector states, we provide a potential classification of the genotype dynamics. We find that the pure cooperator population cannot fixate is not stable in the alternating case.*

*We find that the cooperator homozygote population cannot fixate is not stable in the alternating case.*

What does it mean for a cooperator population to fixate? You mean a mutant cooperator to go to fixation? Also, as I said before, this observation is trivial.

[AUTHORS] We acknowledge that the term "*fixate"* may be misleading in the context of infinite-population deterministic dynamics, as it can suggest a stochastic fixation process. Our original intention was to informally refer to "*evolutionary stability*"—which, in our model, also implies "*asymptotic stability*" in the genotype dynamics. To avoid confusion, we have rephrased the relevant sentences using "*stability*" instead of "*fixation*". We have also systematically reviewed the manuscript and replaced other potentially ambiguous uses of "*fixate*" and "*fixation*" with terminology that clearly refers to stability properties.

As for the observation that the cooperator homozygote population cannot be evolutionarily stable in the alternating (anti-coordinated) case: although this may seem intuitive, it is not entirely trivial. Its verification requires the explicit stability conditions for pure cooperator states provided in Table 2 (derived in the Supplementary Information). Only by comparing these conditions with the definition of the anti-coordinated case does it become clear that the cooperator homozygote population cannot be an ESGD in this scenario.

[REVIEWER]

*However, in the cooperation case, fixation is possible but not necessary, since bistability, coexistence, …*

This is not a sentence. What does this mean? Totally vague.

[AUTHORS] We agree that the sentence lacked clarity and was incomplete. We have revised it to better reflect our intended meaning, namely that in the coordinated case, different dynamical outcomes—such as bistability or interior coexistence—may arise depending on parameter values.

[REVIEWER]

*… moreover, the monostable fixation of the pure defector state can also occur due to the interplay between the phenotypic payoff function and the genotype-phenotype mapping, which collectively determine the outcome of natural selection.*

What does this mean? We are doing infinite population, deterministic dynamics, so why use the word fixation? Why not use terms like stable monomorphic state or something like that. And why leave the interplay a black box? These two ingredients are not both needed in conjunction to get bistability and/or coexistence.

[AUTHORS] As mentioned above, we have replaced *fixation* with *stability* to maintain terminological consistency. We have also rephrased the sentence involving the word *interplay* to be more precise. Our point is that even when the phenotypic payoff matrix is fixed, the structure of the genotype–phenotype mapping (i.e., the inheritance system) can determine whether an interior or boundary rest point becomes stable under the genotype dynamics (eg., $A_4$, $A_5$ in Table 10).

[REVIEWER]

*In the donation game, the classical Hamilton's rule implies the fixation of the cooperation in all considered genotype-phenotype mappings.*

First of all "the cooperation" should be "cooperation". Also, this is a strange way to put this. Hamilton's rule does not imply fixation of cooperation in general, it is a rule that describes when cooperation does and when it does not fixate (again, if that is the teminology used, which I would argue against, because it really staes when cooperation is selected for, and with frequency dependent costs and/or benefits that can allow for stable mixtures). And these are just superficial comments about the language. The abstract also does not really focus on what is most important. I will leave it at this, but the rest of the paper has similar suboptimalities in the language.

[AUTHORS] We have revised the sentence in question to improve clarity. In particular, we have rephrased it and to more accurately reflect the role of Hamilton's rule as a condition under which cooperation is favored by selection.

Regarding your broader remark on the language: we have carefully gone through the entire manuscript to correct linguistic inaccuracies, improve the fluency of the text, and enhance precision throughout. We are grateful for your comments, which helped us identify and address several important issues in the presentation.

[REVIEWER]

Just to comment on something that the authors say in their response, in defense of not clarifying the usefulness of their approach in relation tot the inclusive fitness literature:

*As mentiond, in the previous review, "The field of inclusive fitness can be rather toxic and has some unscientific characteristics." Our previous experiences support this opinion. Therefore, whenever possible, we strictly focus on our population genetic model. If we start arguing in the Introduction in defense of our model—explaining why genotype-centered models are necessary for studying kin selection in diploid populations—we fear that we may lose some potential readers, particularly those who strongly adhere to Hamilton's rule.*

I do of course totally sympathise, but I think the risk-assesment by the authors is wrong. There are two aspects: 1) getting a paper published and 2) getting people to read it. Regarding point 1) you have two sympathetic reviewers, so there is no need to be overly cautious. Regarding point 2) the IF people will not read your paper anyway. Therefore, you should explain to neutral, non-partisan readers why this is helpful, and those are typically not offended by clarity and precision.

[AUTHORS] We appreciate the reviewer's thoughtful comment and understand the encouragement to be more explicit about the usefulness of our approach in relation to the inclusive fitness (IF) literature.

The main reason for adopting a more cautious tone is that our model is formulated purely within the framework of population genetics. In contrast to approaches that rely on the Hardy–Weinberg equilibrium assumption (such as the Fisher model), our model does not build this equilibrium into the dynamics. We have previously shown (Garay & Varga, 2003) that if a diploid population is always in Hardy–Weinberg equilibrium and interactions are well mixed, then the classical ESS concept applies to diploid populations. However, answering the broader question raised by Van Veelen (2009)—namely, When inclusive fitness is right and when it can be wrong.—is difficult and would require an extensive analysis of many different scenarios.

What we can claim more specifically is that within the framework of Haldane's familial selection model, the classical Hamilton's rule generally fails to predict correctly. This is due to a mathematical necessity: at least three parameters (beyond relatedness) are required to characterize the payoff matrices arising in such models. A single cost and benefit parameter, as used in the classical Hamiltonian formulation, is not sufficient. We have emphasized this more clearly in the current version (see lines 771–779).

József Garay, Zoltán Varga, 2003 Coincidence of ESAD and ESS in dominant–recessive hereditary systems, Journal of Theoretical Biology, 222, 297-305,

https://doi.org/10.1016/S0022-5193(03)00027-4.

Van Veelen M (2009) Group selection, kin selection, altruism and cooperation: When inclusive fitness is right and when it can be wrong. J. Theor. Biol., 259: 589–600.
https://doi.org/10.1016/j.jtbi.2009.04.019
